# Supplementary material for: Striatal dopamine dissociates methylphenidate effects on value-based versus surprise-based reversal learning
Source: Nat Commun. 2022 Aug 24;13:4962. doi: 10.1038/s41467-022-32679-1 (PMC9402573; doi:10.1038/s41467-022-32679-1)
Supplement: Supplementary file 1 — Supplementary Information [file 41467_2022_32679_MOESM1_ESM.pdf]

Supplementary information

Table of Contents

Outline of Supplementary Information 2

Supplementary Results 2

Main task effects 2

Dopamine synthesis capacity 4

Striatal dopamine synthesis-dependent effects on striatal BOLD signal 4

Striatal dopamine synthesis-dependent effects on stimulus-specificity in visual cortex 6

Sulpiride increases functional connectivity between caudate nucleus and visual association cortex depending on dopamine synthesis capacity 6

Striatal dopamine synthesis-dependent effect of methylphenidate on prefrontal BOLD signal 7

Main drug effects on outcome-related fMRI BOLD signal 10

Striatal dopamine synthesis-dependent drug effects on reward versus punishment reversal learning 12

Striatal dopamine synthesis-dependent drug effects on reward versus punishment prediction response times 17

BOLD signal predicts behavior 18

Analysis of win-stay/lose-shift behavior 21

Supplementary Discussion 23

Supplementary Methods 25

Performance-based participant exclusion 25

PET acquisition and preprocessing 26

fMRI preprocessing details 27

fMRI quality assurance procedure 28

PPI analysis 29

Analysis of trial-level BOLD signal 29

Brain-behavior correlations 30

Supplementary References 31

Outline of Supplementary Information

In this Supplementary Information document we provide:

- descriptive data of dopamine synthesis capacity and results from validating analyses of general task performance and fMRI effects.
- additional figures and statistics pertaining to breakdowns of significant interaction effects described in the main text. Many figures presented in the main text are median-split to clearly show an effect; in the Supplementary Information, corresponding figures show those effects in a scatter plot version for complete reporting.
- the results of an exploratory generalized psychophysiological interaction (gPPI) analysis investigating drug effects on functional connectivity between brain areas observed in the main results.
- the results of an exploratory trial-by-trial analysis to demonstrate the behavioral relevance of the observed fMRI BOLD signal changes.
- the results of two supplementary control analyses: (i) to correct behavioral effects for age and session number, and (ii) to establish that observed drug effects are not explained by effects on win-stay/lose-shift behavior.

The order of results in the Supplementary Results section follows the order of the Results section in the main text as closely as possible.

Supplementary Results

Main task effects

Across drug sessions, participants were both more accurate and had faster response times (RTs) on trials following an unexpected outcome (reversal trials) than on regular trials (Supplementary Figure 1; Supplementary Table 1; expectancy effect on accuracy: B = 0.217, CI = [0.136, 0.301]; expectancy effect on RT: B = -0.052, CI = [-0.061, -0.043]). The increase in accuracy was larger after unexpected punishments compared with regular punishment trials than it was for rewards (Supplementary Figure 1a; expectancy x valence effect on accuracy: B = -0.055, CI = [-0.105, -0.003]).

Analyses of the main effects of unexpected outcomes on BOLD signal, irrespective of drug session and individual differences, revealed strong task effects, particularly in fronto-striatal circuitry (Figure 1b in main text for main effect of outcome expectancy) comparable with those reported previously[1–3]. In addition, we demonstrate that unexpected outcomes increase face/scene stimulus-specific BOLD signal in visual association cortex, consistent

with the need for updating outcome predictions for faces/scenes following unexpected events (Figure 1c in main text).

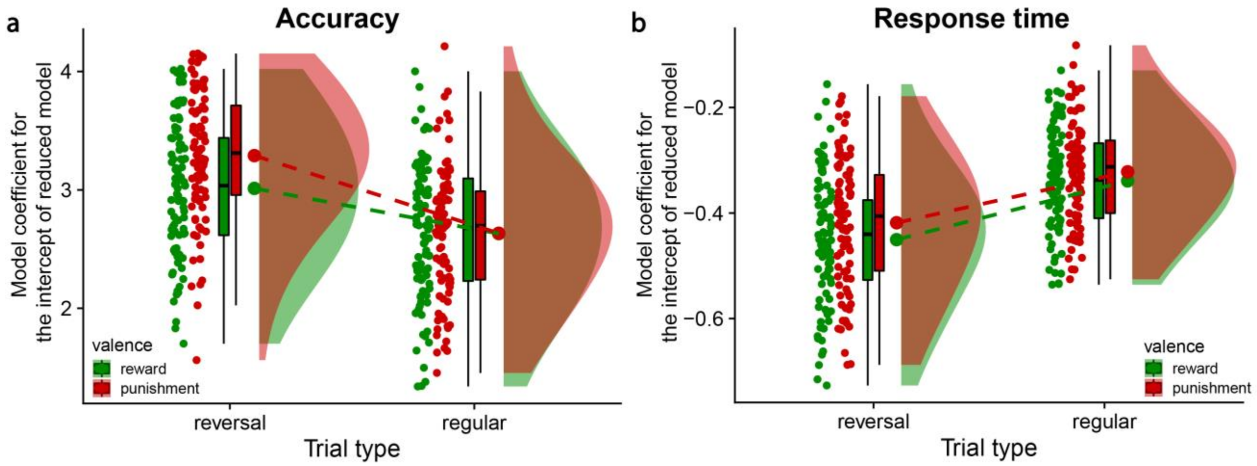

Supplementary Figure 1. ***Across drug sessions, participants were both more accurate and responded faster on reversal trials (after unexpected outcomes) than on regular trials.*** *The increase in accuracy was greater for punishment reversal trials than reward reversal trials.* ***a*** *Model coefficients of the intercepts of reduced models that only contained the trials of each specific condition (e.g. only reward reversal trials). The models contained one factor for drug (methylphenidate, sulpiride, placebo).* ***b*** *Same as in panel A, but the dependent variable was response time. N=88 participants in both panels. Boxplots and round dots next to distribution kernels are defined as in Figure 1c. Source data are provided with this paper.*

Supplementary Table 1. Averaged raw accuracy scores and mean response times (RT) for overall task performance and separated by trial type, for each of the three drug conditions. Values represent mean (standard error of mean) proportion of correct responses and response times in seconds. N=88 participants. Trial types: expected reward (ER), update after unexpected reward (UR), expected punishment (EP), update after unexpected punishment (UP). Source data are provided with this paper. PBO: placebo; MPH: methylphenidate; SUL: sulpiride.

**Drug**	**Overall**		**ER**		**UR**		**EP**		**UP**	
	**accuracy**	**RT**	**accuracy**	**RT**	**accuracy**	**RT**	**accuracy**	**RT**	**accuracy**	**RT**
PBO	0.90 (0.006)	0.740 (0.008)	0.88 (0.007)	0.744 (0.009)	0.92 (0.008)	0.667 (0.011)	0.88 (0.007)	0.754 (0.009)	0.94 (0.008)	0.694 (0.011)
MPH	0.94 (0.005)	0.715 (0.009)	0.93 (0.006)	0.716 (0.009)	0.95 (0.006)	0.639 (0.011)	0.93 (0.005)	0.735 (0.009)	0.96 (0.007)	0.671 (0.012)
SUL	0.88 (0.007)	0.737 (0.007)	0.86 (0.009)	0.741 (0.007)	0.89 (0.010)	0.661 (0.010)	0.87 (0.007)	0.752 (0.008)	0.91 (0.010)	0.685 (0.011)

Dopamine synthesis capacity

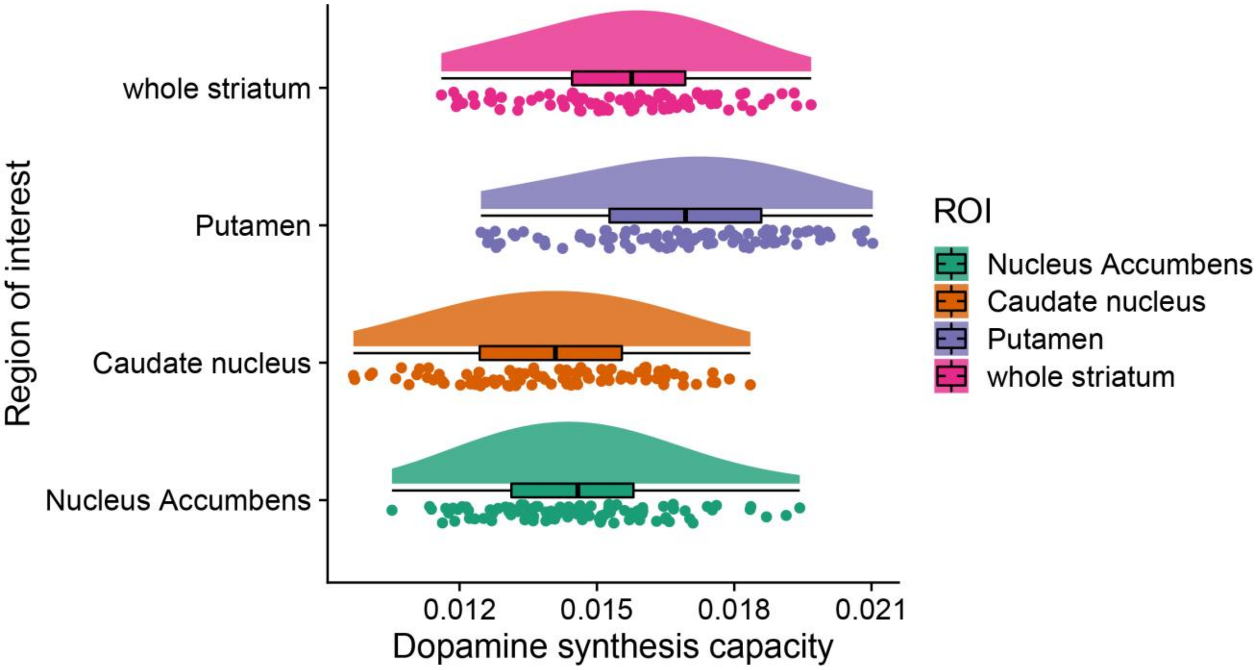

Supplementary Figure 2. ***Distribution of dopamine synthesis capacity (k_i^{cer}) values in the striatal regions of interest (ROI): caudate nucleus, putamen and nucleus accumbens.*** *The whole striatum ROI is the combination of all three subregions of interest. N=94 participants. Boxplots are defined as in Figure 1c. Source data are provided with this paper.*

Supplementary Table 2. Pearson correlation coefficients for dopamine synthesis capacity in the three striatal regions of interest. Source data are provided with this paper.

	Caudate nucleus	Putamen	Nucleus Accumbens
Caudate nucleus	1	0.751	0.649
Putamen	0.751	1	0.787
Nucleus Accumbens	0.649	0.787	1

Striatal dopamine synthesis-dependent effects on striatal BOLD signal

Methylphenidate and sulpiride increased surprise-related BOLD signal in the caudate nucleus to a greater degree in participants with lower dopamine synthesis capacity in the caudate nucleus (Figure 2 main text; Supplementary Figure 3). There was no evidence for an effect of caudate nucleus dopamine synthesis capacity on caudate nucleus BOLD signals

during unexpected outcomes under placebo (Supplementary Figure 4; peak voxel: x,y,z = 10,13,7, Z = 3.50, $p_{peak\ FWE\ SVC}$ = 0.142).

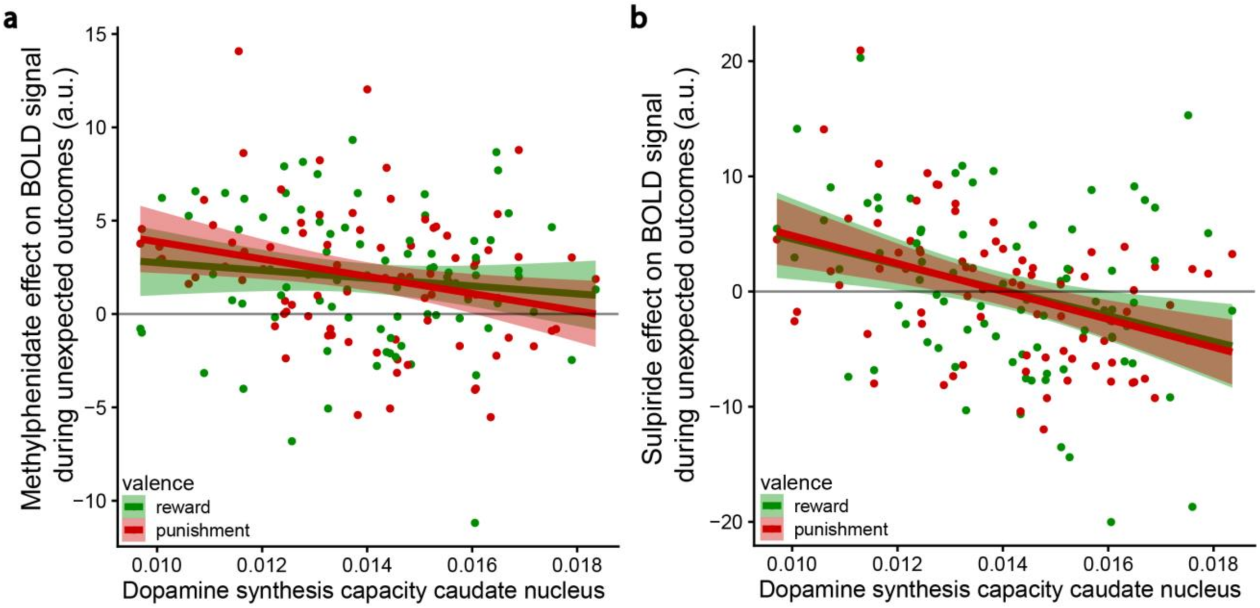

Supplementary Figure 3. ***Drug effects on striatal BOLD signal during unexpected outcomes (relative to placebo).*** *Supplement to Figure 2 main text, without median split.* ***a*** *Effect of methylphenidate on reversal signal in the significant clusters with dopamine synthesis-independent methylphenidate effect (Figure 2a) as a function of caudate nucleus* k_i^{cer}*, displayed separately for unexpected reward and punishment outcomes.* ***b*** *Effect of sulpiride on reversal signal in the significant clusters in the right caudate nucleus (Figure 2c) as a function of caudate nucleus* k_i^{cer}*, displayed separately for unexpected reward and punishment outcomes. Error bands display the 95% confidence interval of the regression lines. Source data are provided with this paper.*

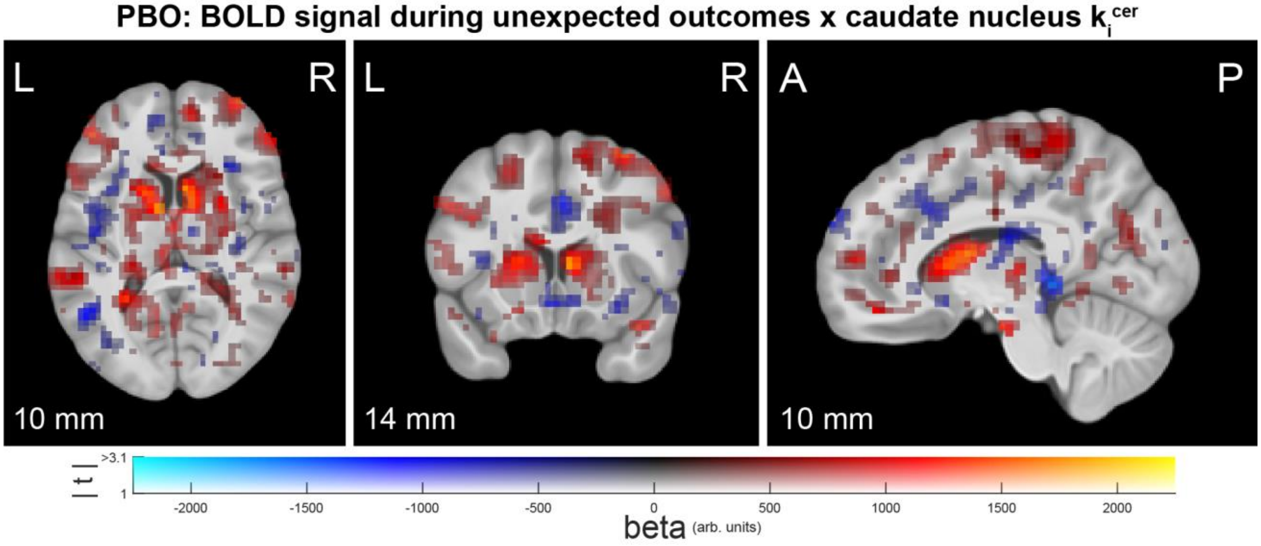

Supplementary Figure 4. ***Data from placebo session.*** *BOLD map showing (absence of) effect of dopamine synthesis capacity in the caudate nucleus on BOLD signal to unexpected outcomes. N=85 participants. Figure conventions are as in Figure 1b. PBO: placebo; arb. units: arbitrary units;* k_i^{cer}*: dopamine synthesis capacity index.*

Striatal dopamine synthesis-dependent effects on stimulus-specificity in visual cortex

There were significant dopamine synthesis-dependent effects of methylphenidate and sulpiride on the stimulus-specificity index of BOLD signal in the FFA/PPA during unexpected outcomes (Figure 3; Supplementary Figure 5). Breakdown of the full interaction revealed that the full interaction for sulpiride was driven by its effect in the FFA compared with the PPA (sulpiride vs placebo x expectancy x caudate nucleus k_i^{cer} in the FFA: $F_{(1,80)}$ = 4.21, p = 0.044; in the PPA: $F_{(1,80)}$ = 1.77, p = 0.187). For methylphenidate the effect was not different in the FFA compared with the PPA, only the difference between the regions was significant (methylphenidate vs placebo x expectancy x caudate nucleus k_i^{cer} in the FFA: $F_{(1,83)}$ = 2.42, p = 0.123; in the PPA: $F_{(1,83)}$ = 2.86, p = 0.094).

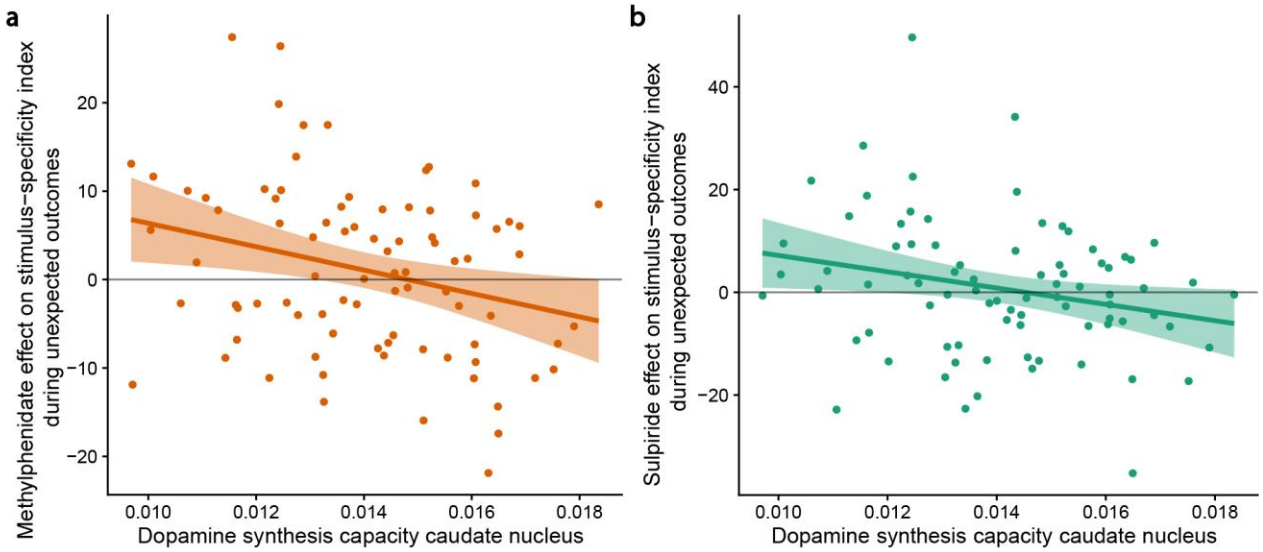

Supplementary Figure 5. ***Drug effects on stimulus-specificity of BOLD signal in the fusiform face area (FFA) and parahippocampal place area (PPA) during unexpected outcomes (relative to placebo).*** *Supplement to Figure 3 main text, without median split. Effect of methylphenidate (**a**) and sulpiride (**b**) on stimulus-specific reversal-related signal in FFA/PPA as a function of caudate nucleus k_i^{cer}. Error bands display the 95% confidence interval of the regression lines. Source data are provided with this paper.*

Sulpiride increases functional connectivity between caudate nucleus and visual association cortex depending on dopamine synthesis capacity

In a between-participants analysis, the dopamine synthesis-dependent effect of methylphenidate on surprising outcome-related signal in the right caudate nucleus correlated significantly with the dopamine synthesis-dependent drug effect on the stimulus-specificity index of FFA/PPA signal during unexpected outcomes (Results in main

text). In a supplementary psychophysiological interaction (PPI) analysis, we tested the relationship between caudate nucleus signal and stimulus-specificity of FFA/PPA signal in a within-participant manner. The analysis tested whether there was significant change in the stimulus-specificity index of FFA/PPA signal as a function of right caudate nucleus activity during unexpected outcomes under methylphenidate or sulpiride compared with placebo, and whether the effect varied as function of dopamine synthesis capacity in the caudate nucleus. It revealed that functional connectivity between the right caudate nucleus and the FFA compared with PPA after unexpected outcomes associated with the face versus the scene stimulus was increased after sulpiride intake, but not methylphenidate intake, for participants with lower compared with higher dopamine synthesis capacity (Supplementary Figure 6; sulpiride x expectancy x caudate nucleus k_i^{cer} effect on FFA/PPA stimulus-specificity index of BOLD signal PPI interaction: $F_{(1,80)} = 7.44$, $p = 0.008$; methylphenidate x expectancy x caudate nucleus k_i^{cer} effect: $F_{(1,83)} = 0.01$, $p = 0.935$). The effect of sulpiride remained significant after excluding the participant with the most extreme outlying negative value ($F_{(1,79)} = 5.57$, $p = 0.019$).

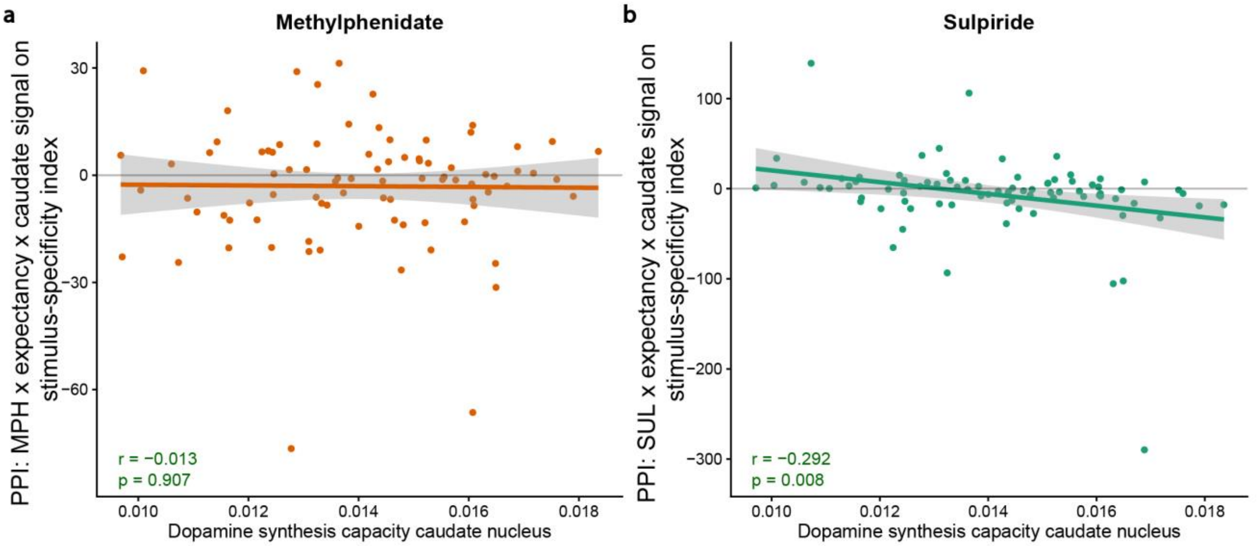

Supplementary Figure 6. ***Drug effects (relative to placebo) on psychophysiological interaction (PPI) between right caudate nucleus signal during unexpected outcomes and the stimulus-specificity of BOLD signal in the fusiform face area (FFA) and parahippocampal place area (PPA).*** *The effects of (**a**) methylphenidate (N=85 participants) and (**b**) sulpiride (N=82) were analyzed as function of dopamine synthesis capacity in the caudate nucleus. Error bands display the 95% confidence interval of the regression lines. Source data are provided with this paper.*

Striatal dopamine synthesis-dependent effect of methylphenidate on prefrontal BOLD signal

Methylphenidate increased prefrontal BOLD signal to unexpected rewards versus punishments as a function of dopamine synthesis capacity in the putamen (Figure 4 main

text) and in the nucleus accumbens (peak voxel with nucleus accumbens k_i^{cer}: x,y,z = -43,39,16, Z = 3.99, $p_{cluster\ FWE\ WB}$ = 0.016). Including three covariates for dopamine synthesis capacity in the analysis of methylphenidate's effect on BOLD signal, one for each striatum ROI, in a single voxel-wise regression model revealed that the bilateral prefrontal clusters were significantly associated with dopamine synthesis capacity in the putamen, over and above the other two regions (Supplementary Figure 7a; peak voxel: x,y,z = 29,63,7, Z = 4.71, $p_{cluster\ FWE\ WB}$ = 0.002).

The putamen dopamine synthesis-dependent effect of methylphenidate in the prefrontal cortex was driven by reward reversal signals and not present for punishment reversal signals (Supplementary Figure 7c; peak voxel in methylphenidate x expectancy x putamen k_i^{cer} for reward only: x,y,z = -20,53,21, Z = 4.69, $p_{cluster\ FWE\ WB}$ = 0.038). Under placebo, reward reversal signals in prefrontal cortex were stronger for 'lower-dopamine' participants, while under methylphenidate, reward reversal signals were stronger for 'higher-dopamine' participants. The negative association under placebo was significant in a voxel-wise regression analysis with dopamine synthesis capacity in the nucleus accumbens, but not putamen (Supplementary Figure 8; prefrontal peak voxel for expectancy x valence x nucleus accumbens k_i^{cer} for placebo only: x,y,z = -47,39,19, Z = 3.74, $p_{cluster\ FWE\ WB}$ = 0.024; prefrontal peak voxel with putamen k_i^{cer} values: x,y,z = 39,49,21, Z = 3.75, $p_{cluster\ FWE\ WB}$ = 0.257).

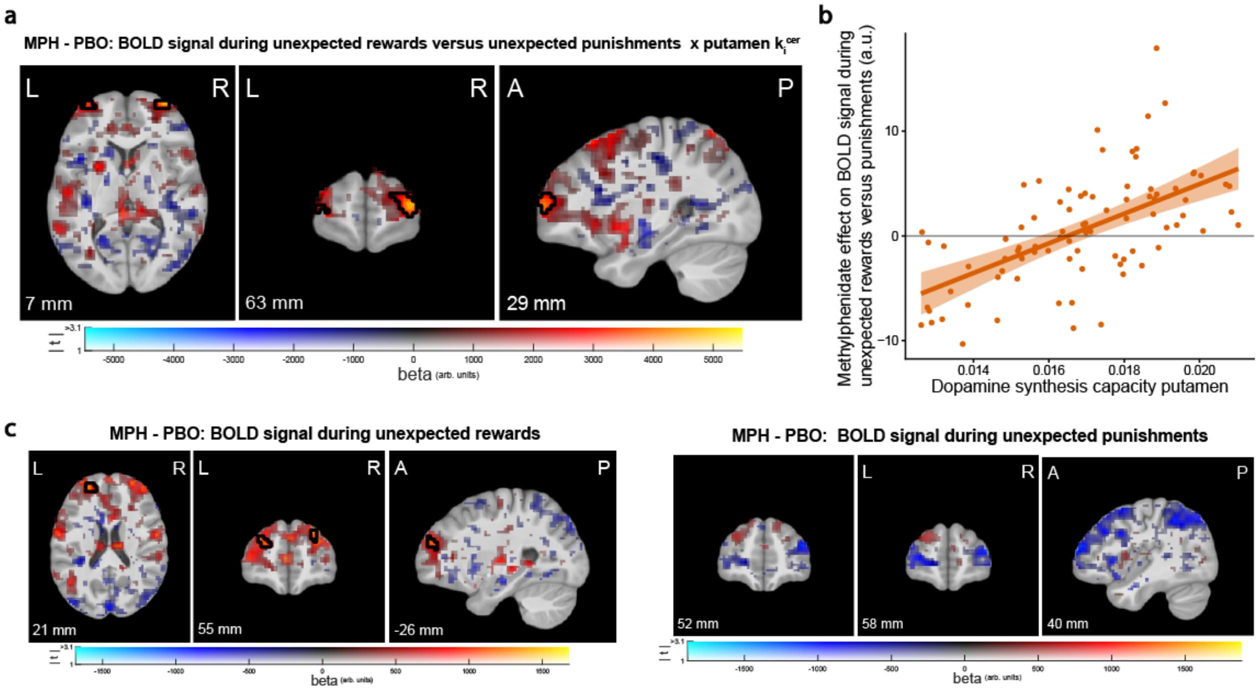

Supplementary Figure 7. ***Methylphenidate effect on prefrontal BOLD signal (relative to placebo). a*** *Methylphenidate increased BOLD signal during unexpected reward versus punishment outcomes in bilateral prefrontal clusters as a function of dopamine synthesis capacity in the putamen, over and above the caudate nucleus and nucleus accumbens. Result for putamen from a general linear model in which* k_i^{cer} *values from all three ROIs were included as covariates.* ***b*** *Average contrast estimates extracted from the significant clusters where methylphenidate increased BOLD signal during unexpected reward versus punishment outcomes as a function of dopamine synthesis capacity in the putamen. Supplement to Figure 4 in main text. Error band displays the 95% confidence interval of the regression line. Source data are provided with this paper.* ***c*** *The putamen dopamine synthesis-dependent effect of methylphenidate in prefrontal cortex was driven by reward reversal signals. There was a significant methylphenidate effect during unexpected rewards, but not during unexpected punishments. Figure conventions are as in Figure 1b. MPH: methylphenidate; PBO: placebo; arb. units: arbitrary units;* k_i^{cer}*: dopamine synthesis capacity index.*

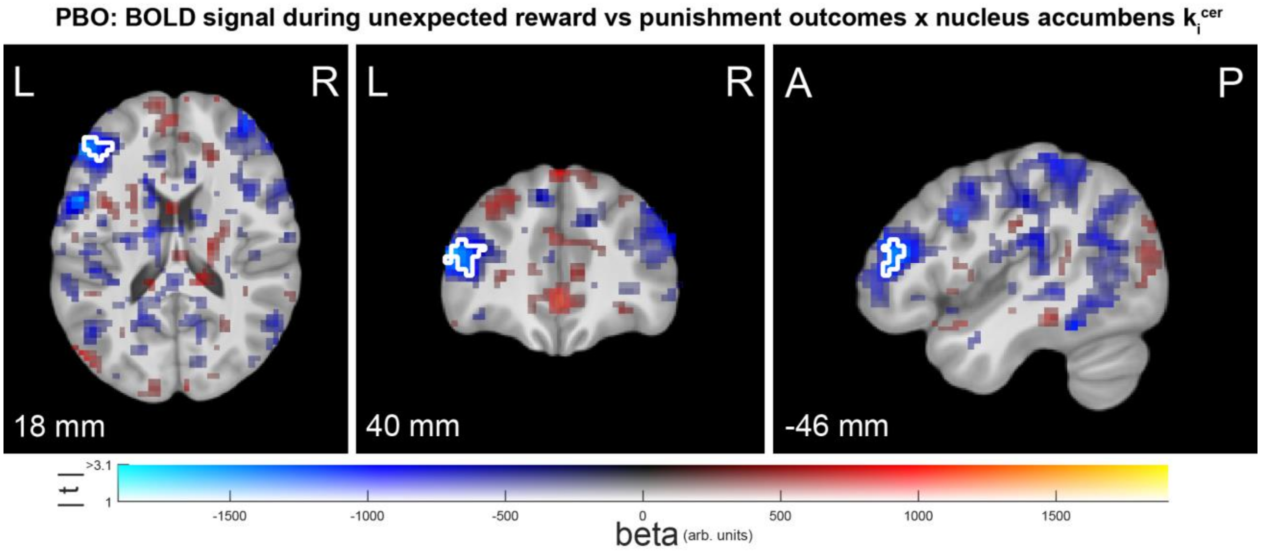

Supplementary Figure 8. ***Data from placebo session.*** *BOLD map showing the negative association between dopamine synthesis capacity in the nucleus accumbens and BOLD signal related to unexpected reward versus unexpected punishment outcomes under placebo. N=85 participants. Figure conventions are as in Figure 1b. PBO: placebo; arb. units: arbitrary units;* k_i^{cer}*: dopamine synthesis capacity index.*

Main drug effects on outcome-related fMRI BOLD signal

Across drug sessions and trial types there were large-scale BOLD signal increases in striatum, motor cortex and posterior parietal cortex, and large-scale BOLD signal decreases in insula, medial frontal cortex, and medial parietal cortex (Supplementary Figure 9a).

Methylphenidate increased BOLD signal across outcome types in bilateral parietal cortex, and this effect was greater in participants with lower dopamine synthesis capacity in all three striatal ROIs (Supplementary Figure 9b-c; Supplementary Table 3). Sulpiride decreased outcome-related BOLD signal across trial types, independent of dopamine synthesis capacity, in the right posterior putamen, and increased BOLD signal in the right motor cortex (Supplementary Figure 9d; peak voxel putamen: x,y,z = 29,-7,-8, Z = 4.72, $p_{peak\ FWE\ SVC} = 0.001$; Supplementary Table 3).

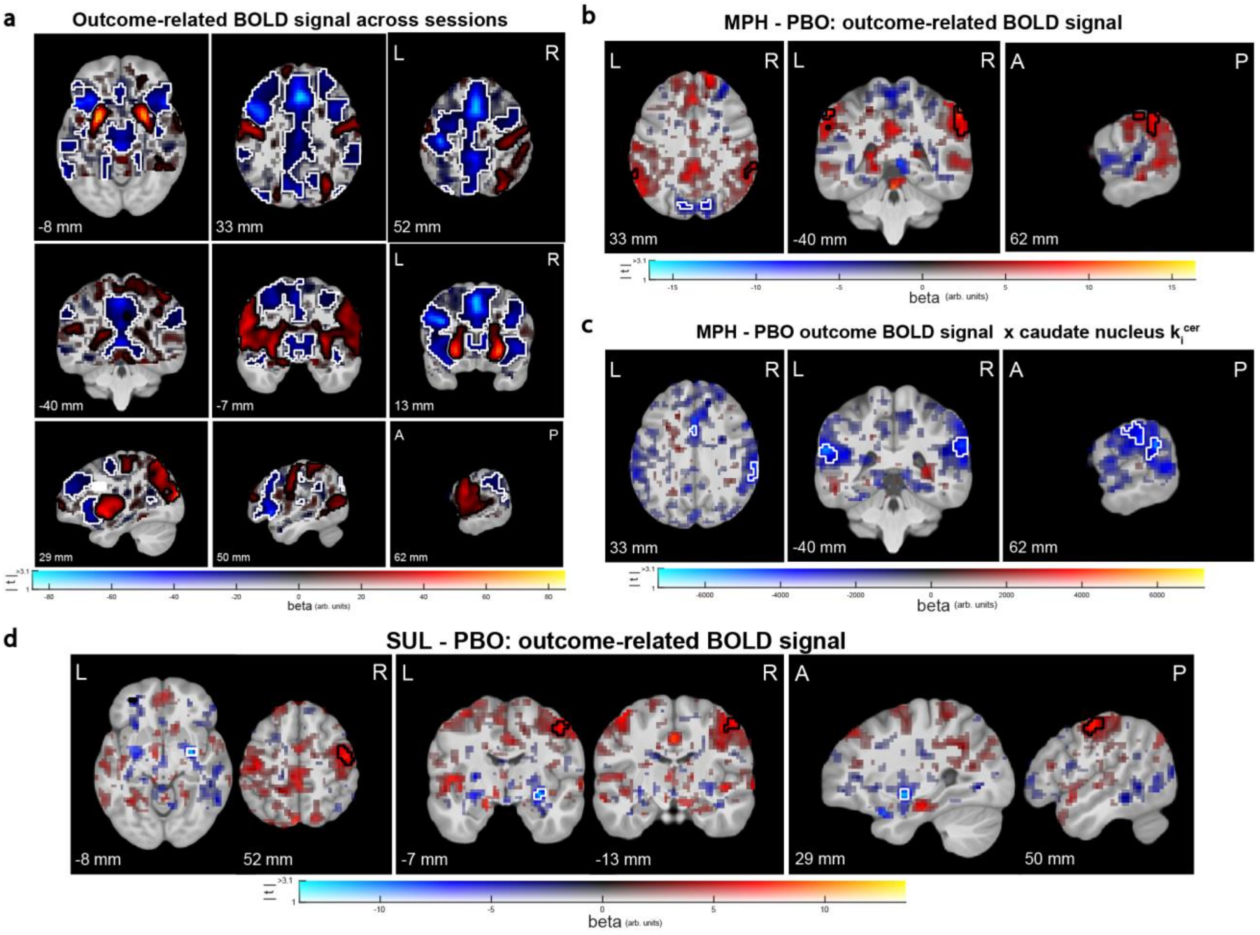

Supplementary Figure 9. ***Main effects on fMRI BOLD signal across outcome types.*** ***a*** *Outcome-related BOLD signal averaged across drug sessions and outcome types, independent of dopamine synthesis capacity.* ***b*** *Main effect of methylphenidate on outcome-related BOLD signal independent of dopamine synthesis capacity, and* ***c*** *as function of dopamine synthesis capacity in the caudate nucleus.* ***d*** *Main effect of sulpiride on outcome-related BOLD signal, independent of dopamine synthesis capacity. Figure conventions are as in Figure 1b. N=94 participants. MPH: methylphenidate; SUL: sulpiride; PBO: placebo; arb. units: arbitrary units;* k_i^{cer}*: dopamine synthesis capacity index.*

Supplementary Table 3. List of all clusters in the one-sample t-test fMRI analyses that were significant at p < 0.001 uncorrected and/or p < 0.05 with cluster-level FWE correction at the whole-brain level. MPH: methylphenidate; SUL: sulpiride; PBO: placebo; BA: Brodmann Area; aPFC: anterior prefrontal cortex.

Contrast	Direction of effect	Region	hemisphere	x	y	z	voxel Z-value	cluster size (k)	p(k) uncorrected	p(k) FWE correction
MPH main effect	positive	supramarginal gyrus / BA 40	L	-60	-43	48	4	28	0.003	0.046
MPH main effect	positive	supramarginal gyrus / BA 40	R	62	-40	33	3.88	54	1.08e-04	0.002
MPH main effect	positive	supramarginal gyrus / BA 40	L	-56	-63	45	3.85	46	2.72e-04	0.005
MPH main effect	negative	superior occipital lobe / BA 7	L	-14	-79	27	5.14	59	6.19e-05	0.001
MPH main effect	negative	superior occipital lobe / BA 7	R	13	-79	33	4.12	29	0.002	0.04
MPH main effect	negative	motor cortex M1 / BA 4	R	33	-23	62	4.06	44	3.46e-05	0.006
MPH main effect x putamen k_i^{cer}	negative	Insula / BA 13	L	-43	0	-11	4.51	28	0.003	0.046
MPH main effect x putamen k_i^{cer}	negative	dorsal posterior cingulate / BA 31	L	0	-17	45	4.37	28	0.003	0.046
MPH main effect x putamen k_i^{cer}	negative	supramarginal gyrus / BA 40	R	69	-36	30	4.05	52	1.35e-04	0.002
MPH main effect x caudate k_i^{cer}	negative	supramarginal gyrus / BA 40	L	-60	-33	21	4.55	108	4.78e-07	8.18e-06
MPH main effect x caudate k_i^{cer}	negative	dorsal posterior cingulate / BA 31	L	0	-17	45	4.37	34	0.001	0.02
MPH main effect x caudate k_i^{cer}	negative	supramarginal gyrus / BA 40	R	59	-43	30	4.34	110	4.00e-08	6.83e-06

MPH main effect x caudate k_i^{cer}	negative	inferior frontal gyrus	R	46	6	19	4.18	48	2.08e-04	0.004
MPH main effect x accumbens k_i^{cer}	negative	somatosensory cortext / BA 1	R	66	-20	30	3.72	33	0.001	0.023
MPH main effect x accumbens k_i^{cer}	negative	ventral anterior cingulate cortex (ACC)	L	-4	0	39	3.71	36	0.001	0.016
MPH x expectancy	positive	Putamen	L	-30	3	-5	4.34	72	2.02e-05	3.32e-04
MPH x expectancy x valence x putamen k_i^{cer}	positive	aPFC / BA 10	L	-20	59	-2	5.25	42	3.65e-04	0.006
MPH x expectancy x valence x putamen k_i^{cer}	positive	dlPFC / BA 46	R	39	53	21	4.23	27	0.003	0.047
MPH x expectancy x valence x accumbens k_i^{cer}	positive	dlPFC / BA 46	L	-43	39	16	3.99	35	0.001	0.016
SUL main effect	positive	motor cortex M1 / BA 4	R	52	-13	54	4.38	54	1.59e-04	0.003
PBO: main effect of caudate k_i^{cer}	positive	supramarginal gyrus / BA 40	L	-63	-30	21	4.86	35	0.001	0.021
PBO: expectancy x caudate k_i^{cer}	positive	somatosensory cortex / BA 5	L	0	-33	59	4.74	102	1.95e-06	3.04e-05
PBO: expectancy x valence x accumbens k_i^{cer}	negative	premotor cortex/BA 6	L	-20	-13	62	4.24	36	0.001	0.016
PBO: expectancy x valence x accumbens k_i^{cer}	negative	aPFC / BA 46	L	-47	39	19	3.74	33	0.001	0.024

Striatal dopamine synthesis-dependent drug effects on reward versus punishment reversal learning

Breakdown of the significant dopamine synthesis-dependent effect of methylphenidate on reward versus punishment reversal accuracy into its constituent simple interaction effects revealed that the effect was primarily driven by dopamine synthesis-dependent modulation of punishment learning: methylphenidate boosted punishment reversal accuracy to a greater degree in participants with lower striatal dopamine synthesis capacity (Figure 5a main text; Supplementary Figure 10a; methylphenidate x expectancy x putamen k_i^{cer} for punishment: B = -0.131, CI = [-0.226, -0.037]; methylphenidate x putamen k_i^{cer} for punishment reversal trials: B = -0.222, CI = [-0.417, -0.026]; methylphenidate x putamen k_i^{cer} for punishment regular trials: B = 0.028, CI = [-0.042, 0.098]; methylphenidate x expectancy x putamen k_i^{cer} for reward: B = 0.007, CI = [-0.076, 0.088]). Further breakdown revealed a significant negative effect of putamen k_i^{cer} on punishment reversal accuracy under methylphenidate (B = -0.328, CI = [-0.686, -0.014]). There was no evidence for an effect of putamen k_i^{cer} on punishment reversal accuracy under placebo (B = 0.093, CI = [-0.187, 0.384]).

The significant dopamine synthesis-dependent effect of sulpiride was primarily driven by changes in reward rather than punishment learning (Figure 5c main text; Supplementary Figure 10b; sulpiride x expectancy x caudate nucleus k_i^{cer} for reward: B = 0.086, CI = [0.017, 0.156]; sulpiride x caudate nucleus k_i^{cer} for reward reversal trials: B = 0.130, CI = [-0.005, 0.274]; sulpiride x caudate nucleus k_i^{cer} for reward regular trials: B = -0.058, CI = [-0.117, 0.000]; sulpiride x expectancy x caudate nucleus k_i^{cer} for punishment: B = -0.059, CI = [-0.140, 0.022]). There was no evidence for lower-order interaction effects (effect of caudate nucleus k_i^{cer} on reward reversal accuracy under placebo: B = -0.072, CI = [-0.344, 0.187]; and under sulpiride (B = 0.213, CI = [-0.021, 0.448]; effect of caudate nucleus k_i^{cer} on punishment reversal accuracy under placebo: B = 0.259, CI = [-0.025, 0.547] and sulpiride: B = -0.037, CI = [-0.296, 0.224]).

Given the association between age and striatal dopamine function, as measured with [^{18}F]DOPA PET[4], we assured that the synthesis-dependent effects of methylphenidate and sulpiride on reward versus punishment learning were not explained by this confounding factor. In addition, we assured that the effect was also not explained by practice effects of repeatedly performing the task over three drug sessions. To this end, we included age and session number as additional predictors in the Bayesian mixed-effects models, both as main effects as well as in interaction with the other factors. The factor session was included in both the fixed and random effects, while the between-participants variable age was only included in the fixed effects. These analyses revealed that age and session number did not

affect the significant synthesis-dependent drug effects on reward versus punishment reversal accuracy (Supplementary Table 4).

In contrast to prior PET work with this task[5], there was a negative effect of dopamine synthesis capacity under placebo. Reward versus punishment reversal accuracy was lower for participants with higher caudate nucleus dopamine synthesis capacity (Supplementary Figure 11).

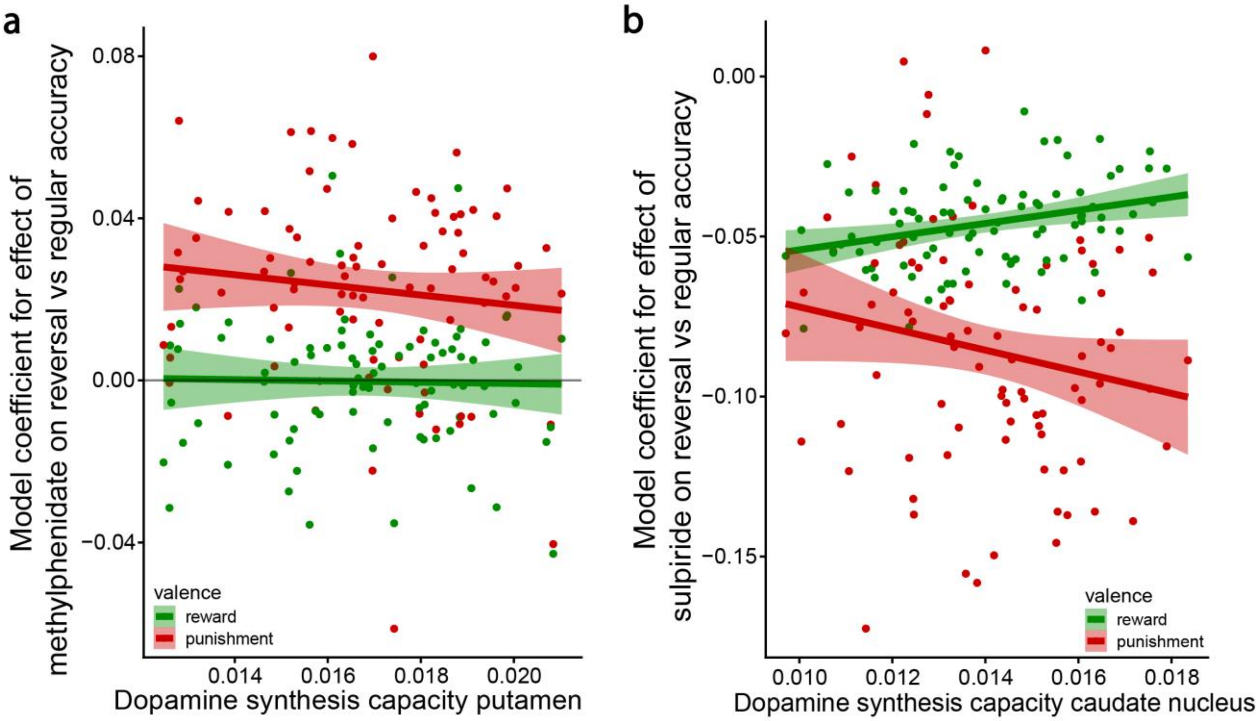

Supplementary Figure 10. ***Relationship between dopamine synthesis capacity and drug effects on reward versus punishment reversal learning performance (relative to placebo).*** *Supplement to Figure 5 in main text.* ***a*** *Methylphenidate increased punishment versus reward reversal accuracy to a greater degree in participants with lower striatal dopamine synthesis capacity. Model coefficients for the effect of methylphenidate on reward and punishment reversal accuracy as function of putamen* k_i^{cer}*.* ***b*** *Sulpiride increased reward versus punishment reversal accuracy to a greater degree in participants with higher striatal dopamine synthesis capacity. Model coefficients for the effect of sulpiride on reward and punishment reversal accuracy as function of caudate nucleus* k_i^{cer}*. Error bands display the 95% confidence interval of the regression lines. Source data are provided with this paper.*

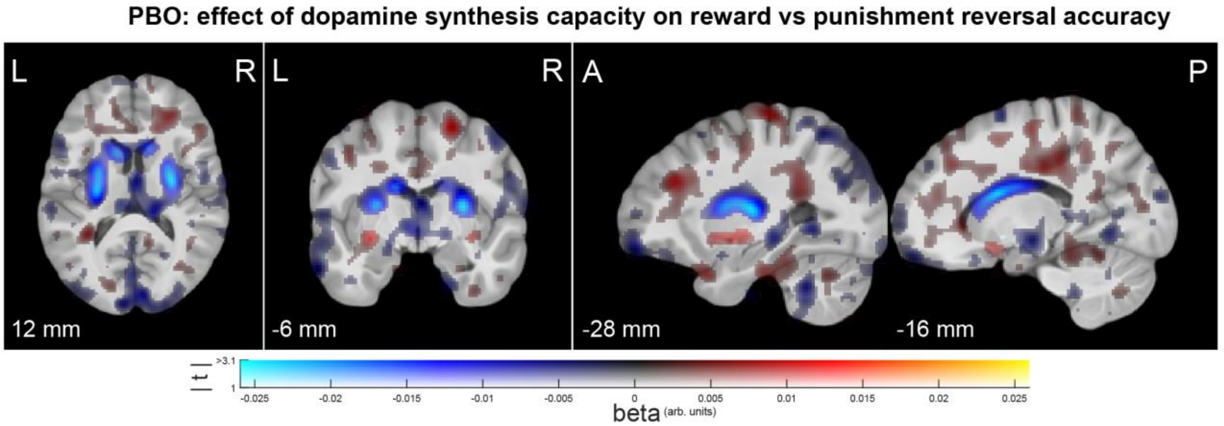

Supplementary Figure 11. ***Relationship between striatal dopamine synthesis capacity and the effect of unexpected rewards versus unexpected punishments on accuracy under placebo.*** *Voxel-wise covariation analysis of the PET k_i^{cer} data with individual participants' model coefficients for the expectancy x valence interaction effect on accuracy as covariate. N=88 participants. Figure conventions are as in Figure 1b. PBO: placebo; arb. units: arbitrary units.*

Supplementary Table 4. Most important drug effect estimates on accuracy. The Bayesian mixed-effects model estimates and credible intervals of the effects are presented from the original models and from models including the confound variables age and session number. CI_lower: lower bound of the 95% credible interval; CI_upper: upper bound of the 95% credible interval; MPH: methylphenidate; SUL: sulpiride; k_i^{cer}: dopamine synthesis capacity index.

effect	original accuracy models			extra accuracy models		
	estimate	CI_lower	CI_upper	estimate	CI_lower	CI_upper
MPH main effect	0.2736	0.1904	0.3573	0.2799	0.2016	0.3591
SUL main effect	-0.1346	-0.2065	-0.0664	-0.1206	-0.1905	-0.0524
MPH x expectancy x valence x putamen k_i^{cer}	0.0654	0.0050	0.1254	0.0718	0.0076	0.1383
SUL x expectancy x valence x caudate nucleus k_i^{cer}	0.0703	0.0194	0.1218	0.0690	0.0167	0.1212
MPH x expectancy x valence x age				-0.0268	-0.1015	0.0459
SUL x expectancy x valence x age				-0.0225	-0.0883	0.0424
session x expectancy x valence x putamen k_i^{cer}				-0.0514	-0.1316	0.0287
session x expectancy x valence x caudate nucleus k_i^{cer}				-0.0142	-0.0803	0.0513

Striatal dopamine synthesis-dependent drug effects on reward versus punishment prediction response times

The significant interaction effects of methylphenidate and sulpiride speeding response times for punishment versus reward predictions in participants with lower dopamine synthesis capacity (Figure 6 main text; Supplementary Figure 12) were broken down in their constituent parts to test whether the effects were driven by reward or punishment trials. The simple interaction effects for reward or punishment trials separately were not significant (methylphenidate x nucleus accumbens k_i^{cer} for reward trials: B = 0.002, CI = [-0.011, 0.015]; for punishment trials: B = 0.011, CI = [-0.001, 0.022]; sulpiride x putamen k_i^{cer} for reward trials: B = -0.003, CI = [-0.012, 0.008]; for punishment trials: B = 0.009, CI = [-0.001, 0.019]; sulpiride x nucleus accumbens k_i^{cer} for reward trials: B = -0.008, CI = [-0.017, 0.002]; for punishment trials: B = 0.005, CI = [-0.005, 0.015]).

Including age and session number as additional predictors in the Bayesian mixed-effects models as a control analysis revealed that these factors did not affect the significant synthesis-dependent effect of sulpiride on reward versus punishment prediction RTs (Supplementary Table 5). In these control models with additional variables, the effect of methylphenidate was no longer significant, however there were also no significant effects of age or session number of reward versus punishment prediction RTs (methylphenidate x valence x nucleus accumbens k_i^{cer} on RTs: B = -0.003, CI = [-0.007, 0.000]; methylphenidate x valence x age: B = 0.000, CI = [-0.004, 0.004]; session x valence x nucleus accumbens k_i^{cer}: B = -0.001, CI = [-0.006, 0.004]).

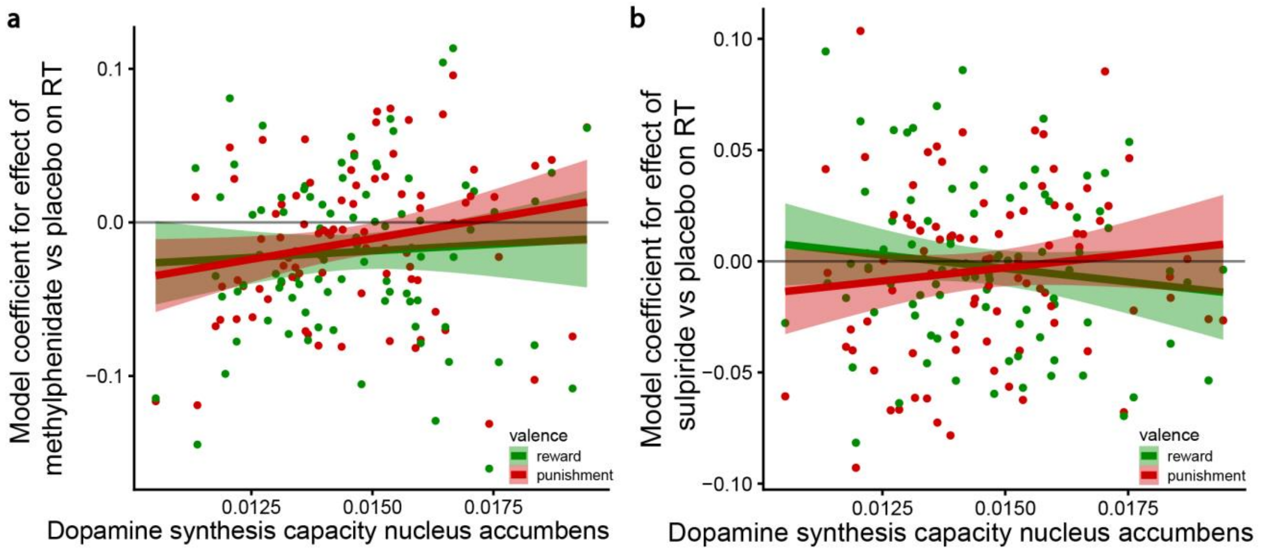

Supplementary Figure 12. ***Relationship between dopamine synthesis capacity and drug effects (relative to placebo) on reward versus punishment prediction response times (RTs).*** *Supplement to Figure 6 in main text. Methylphenidate (**a**) and sulpiride (**b**) decreased punishment versus reward prediction RTs to a greater*

degree in participants with lower striatal dopamine synthesis capacity. Model coefficients for the effects of methylphenidate and sulpiride on reward and punishment prediction RT as function of nucleus accumbens k_i^{cer}. Error bands display the 95% confidence interval of the regression lines. Source data are provided with this paper.

Supplementary Table 5. Most important drug effect estimates on response times. The Bayesian mixed-effects model estimates and credible intervals of the effects are presented from the original models and from models including the confound variables age and session number. RT: response time; CI_lower: lower bound of the 95% credible interval; CI_upper: upper bound of the 95% credible interval; MPH: methylphenidate; SUL: sulpiride; k_i^{cer}: dopamine synthesis capacity index.

effect	original RT models			extra RT models		
	estimate	CI_lower	CI_upper	estimate	CI_lower	CI_upper
MPH main effect	-0.0162	-0.0279	-0.0050	-0.0139	-0.0229	-0.0044
SUL main effect	-0.0032	-0.0122	0.0061	-0.0004	-0.0094	0.0083
MPH x valence x nucleus accumbens k_i^{cer}	-0.0043	-0.0078	-0.0009	-0.0035	-0.0074	0.0005
SUL x valence x nucleus accumbens k_i^{cer}	-0.0064	-0.0097	-0.0030	-0.0056	-0.0093	-0.0018
MPH x valence x age				0.0002	-0.0039	0.0041
SUL x valence x age				0.0031	-0.0008	0.0070
session x valence x nucleus accumbens k_i^{cer}				-0.0011	-0.0062	0.0039

BOLD signal predicts behavior

The behavioral relevance of the observed BOLD signal changes was evidenced by exploratory Bayesian mixed effects modeling of accuracy and RT at the trial-by-trial level and with between-participants correlations.

Higher trial-level estimates outcome-related BOLD signal in the aPFC and putamen clusters, as well as greater stimulus-specificity of FFA/PPA signal, significantly predicted higher accuracy on the subsequent trial (main effect of aPFC signal: B = 0.071, CI = [0.026, 0.115]; main effect of putamen signal: B = 0.063, CI = [0.016, 0.108]; main effect of stimulus-specificity FFA/PPA signal: B = 0.050, CI = [0.004, 0.096]). Higher outcome-related signal in the aPFC and putamen, but not stimulus-specificity in FFA/PPA, also significantly predicted faster subsequent RTs (negative main effect of previous-outcome aPFC signal on RT: B = -0.007, CI = [-0.009, -0.004]; putamen signal: B = -0.004, CI = [-0.007, -0.001]; main effect of stimulus-specificity FFA/PPA signal: B = -0.001, CI = [-0.003, 0.002]). The associations with

performance for these regions did not vary with outcome expectancy or valence. In contrast to the putamen signal, higher previous-outcome signal in the right caudate nucleus cluster displaying the significant methylphenidate effect, but not the cluster with the sulpiride effect (Figures 2b-c), predicted lower subsequent accuracy (main effect of previous-outcome signal in methylphenidate's caudate nucleus cluster on accuracy: B = -0.052, CI = [-0.096, -0.006]; sulpiride's caudate nucleus cluster: B = -0.024, CI = [-0.069, 0.020]). The interaction effects of caudate nucleus signal with expectancy, as well as expectancy and valence, on accuracy were positive, but not significant (methylphenidate's cluster x expectancy: B = 0.026, CI = [-0.019, 0.072]; methylphenidate's cluster x valence: B = -0.001, CI = [-0.054, 0.036]; methylphenidate's cluster x expectancy x valence: B = 0.009, CI = [-0.035, 0.053]; sulpiride's cluster x expectancy: B = 0.033, CI = [-0.010, 0.077]; sulpiride's cluster x valence: B = -0.014, CI = [-0.060, 0.032]; sulpiride's cluster x expectancy x valence: B = 0.006, CI = [-0.038, 0.050]). Outcome-related signal in both of the caudate nucleus clusters significantly predicted slower subsequent RTs (positive main effect of previous-outcome signal in methylphenidate's cluster on RT: B = 0.006, CI = [0.003, 0.008]; sulpiride's caudate nucleus cluster: B = 0.006, CI = [0.004, 0.009]).

The regional specificity of the effects on accuracy was evidenced by a control analysis with outcome-related BOLD signal in the bilateral supramarginal gyrus, where methylphenidate significantly increased activity to all outcomes (Supplementary Table 3). Outcome-related signal in this region did not predict accuracy on the subsequent trial (main effect on accuracy: B = -0.018, CI = [-0.064, 0.028]), but higher signal did predict faster subsequent RTs (B = -0.007, CI = [-0.009, -0.004]).

The between-participants brain-behavior correlations revealed that larger sulpiride-induced increases in reversal signal in the right caudate nucleus were associated with greater punishment versus reward reversal accuracy (i.e. a negative correlation with reward versus punishment reversal accuracy: r = -0.272, p = 0.014; Supplementary Figure 13). A similar association for methylphenidate was not significant (r = -0.146, p = 0.188). There were no further significant associations with model coefficients of accuracy (Supplementary Table 6).

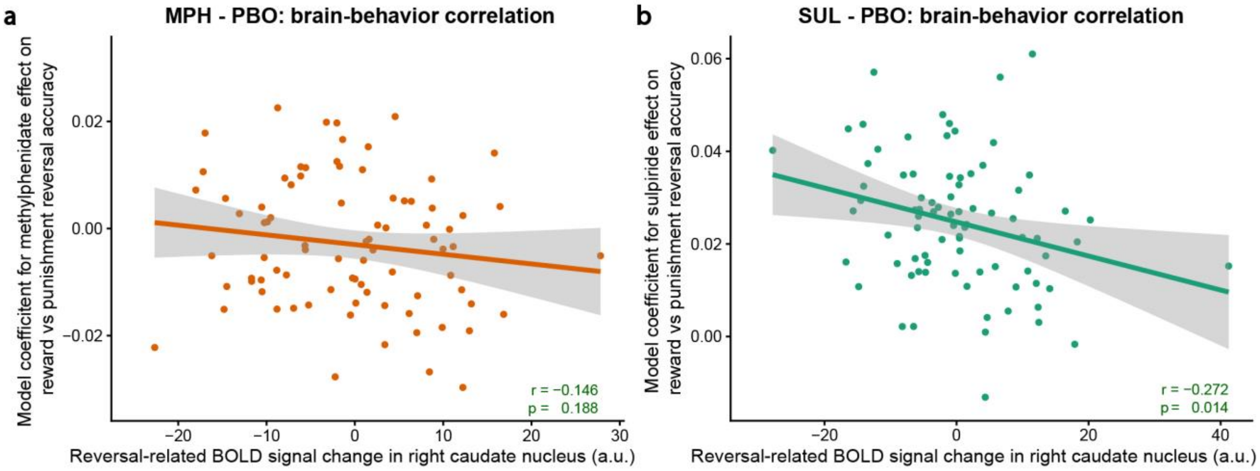

Supplementary Figure 13. ***Between-participants brain-behavior correlations.*** *Correlation between the effect of (**a**) methylphenidate and (**b**) sulpiride (relative to placebo) on reversal-related BOLD signal in the right caudate nucleus and the model coefficient for the effect of methylphenidate/sulpiride on reward versus punishment reversal accuracy (N=85 participants for methylphenidate; N=82 for sulpiride). MPH: methylphenidate; SUL: sulpiride; PBO: placebo. Error bands display the 95% confidence interval of the regression lines. Source data are provided with this paper. arb. units: arbitrary units.*

Supplementary Table 6. Correlations between individual contrast estimates from the statistically significant clusters in group-level contrasts and model coefficients for three behavioral effects on accuracy and response time: main drug effect, drug x reversal (reversal versus regular trials) interaction, drug x reversal x valence (reward versus punishment) interaction. r: two-sided Pearson correlation coefficient. p: uncorrected p-value of the correlation coefficient. Source data are provided with this paper.

**fMRI contrast**	**Model coefficient for accuracy**						**Model coefficient for response time**					
	**main drug effect**		**drug x reversal**		**drug x reversal x valence**		**main drug effect**		**drug x reversal**		**drug x reversal x valence**	
	**r**	**p**	**r**	**p**	**r**	**p**	**r**	**p**	**r**	**p**	**r**	**p**
Methylphenidate x expectancy x caudate nucleus k_i^{cer} in right caudate nucleus	0.073	0.510	0.180	0.103	-0.146	0.188	-0.061	0.586	-0.029	0.792	-0.109	0.329
sulpiride x expectancy x caudate nucleus k_i^{cer} in right caudate nucleus	0.207	0.063	0.128	0.255	-0.272	0.014	-0.140	0.213	-0.109	0.333	0.087	0.440
methylphenidate x expectancy x valence x putamen k_i^{cer} in aPFC	-0.154	0.165	0.055	0.622	0.042	0.708	0.059	0.599	0.002	0.988	-0.069	0.535
methylphenidate x expectancy x caudate nucleus k_i^{cer} on stimulus-specificity in FFA/PPA	0.018	0.876	0.021	0.854	0.003	0.978	0.234	0.039	0.250	0.028	-0.013	0.913
sulpiride x expectancy x caudate nucleus k_i^{cer} on stimulus-specificity in FFA/PPA	0.103	0.375	-0.028	0.813	-0.198	0.087	0.077	0.507	0.093	0.425	-0.040	0.730
Methylphenidate x expectancy in putamen	0.126	0.275	0.097	0.385	0.047	0.674	0.033	0.764	0.030	0.790	0.157	0.156

Analysis of win-stay/lose-shift behavior

The outcome contingencies in this task are counter-intuitive. In most real-life situations, rewards signal correct behavior and punishments signal the need for adjustment of behavior. This results in a tendency to adopt a win-stay/lose-shift response strategy, i.e. to repeat responses after rewards and to switch response after punishments. In the current task such a win-stay/lose-shift strategy is inappropriate, because rewards and punishments

are not contingent on the response that participants made preceding the outcome receipt, but rather on which stimulus was highlighted.

We considered the possibility that participants might nevertheless have adopted a task-inappropriate win-stay/lose-shift strategy, and we asked whether the effect of methylphenidate might reflect modulation of such a strategy. Given the fact that in our design both reward and punishment reversal trials always required response shifting, it is possible that the striatal dopamine synthesis-dependent boosting of punishment reversal accuracy by methylphenidate reflects a potentiation of a win-stay/lose-shift tendency. To investigate whether adoption of that strategy explains our results, we modeled the probability of staying with the same response to the face/scene stimulus as the previous time that stimulus was highlighted.

We found that methylphenidate boosted the probability of staying with the same response after reward versus punishment outcomes to a greater degree in participants with lower dopamine synthesis capacity in the putamen (methylphenidate x valence x putamen k_i^{cer}: B = -0.060, CI = [-0.118, -0.003]). This effect of methylphenidate was driven by striatal dopamine synthesis-dependent increase in switch behavior after punishments (methylphenidate x putamen k_i^{cer} for punishment trials: B = 0.112, CI = [0.026, 0.201]; methylphenidate x putamen k_i^{cer} for reward trials: B = -0.001, CI = [-0.080, 0.076]). Methylphenidate increased lose-shift behavior in participants with lower compared with higher dopamine synthesis capacity. However, the effect was not significant when only considering regular non-reversal trials (Supplementary Figure 14a; B = -0.008, CI = [-0.031, 0.014]). Thus, there is no evidence that methylphenidate affected non-reversal win-stay/lose-shift behavior.

The effect of sulpiride on the probability of staying with the same response after reward versus punishment outcomes was significantly qualified by the reversal trials. The drug increased the relative win-stay/lose-shift behavior on reversal trials in participants with lower dopamine synthesis capacity in the caudate nucleus and putamen (sulpiride x expectancy x valence x caudate nucleus k_i^{cer}: B = -0.061, CI = [-0.113, -0.010]; with putamen k_i^{cer}: B = -0.054, CI = [-0.106, -0.004]). Thereby it captures the same effect as the models for reward versus punishment reversal accuracy, since the highlighted stimulus on reversal trials was always the same as on the preceding trial. The effect was not significant when only considering regular non-reversal trials (Supplementary Figure 14b; sulpiride x valence x caudate nucleus k_i^{cer} with only non-reversal trials: B = -0.005, CI = [-0.028, 0.017]; with putamen k_i^{cer}: B = 0.012, CI = [-0.011, 0.034]). Thus, there is also no evidence that sulpiride affected non-reversal win-stay/lose-shift behavior.

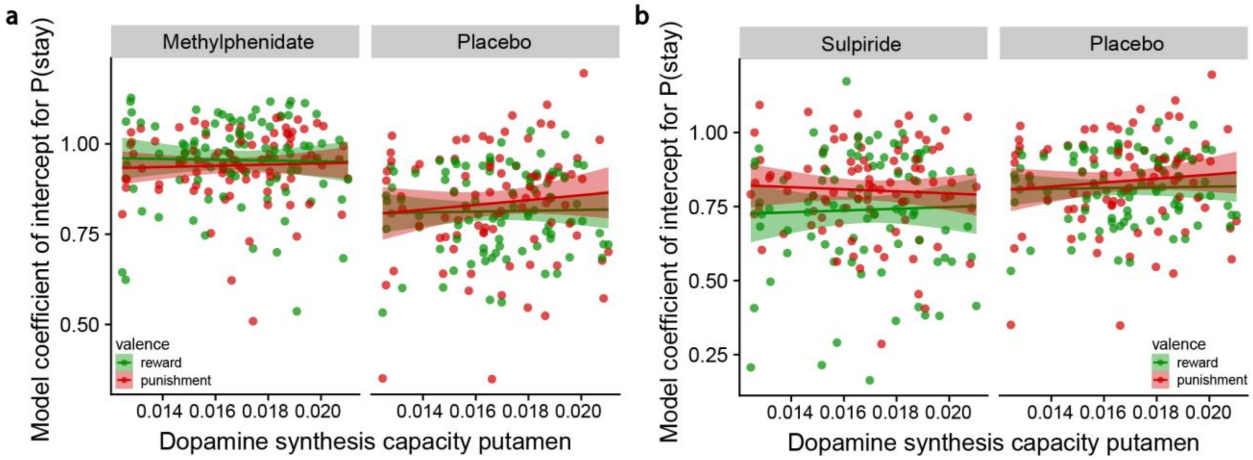

Supplementary Figure 14. ***No drug effects on win-stay/lose-shift behavior on non-reversal trials.*** *Correlation between dopamine synthesis capacity in the putamen and the model coefficients for the intercept of reduced models of the probability to stay with the same response that only included non-reversal reward or punishment trials, displayed separately for the methylphenidate (**a**) or sulpiride (**b**) and placebo sessions. N=88 participants. Error bands display the 95% confidence interval of the regression lines. Source data are provided with this paper.*

Supplementary Discussion

There were significant main effects of outcome-related BOLD signal on the accuracy and RT on subsequent trials in the trial-by-trial, within-participants analyses. These revealed that greater activity in the aPFC and putamen, and greater stimulus-specificity of FFA/PPA signal, was associated with better and faster task performance. It links the observations in the fMRI brain data that methylphenidate increased activity in these regions during surprising outcomes with the observations in the behavioral data that the drug improved performance on the subsequent trial. However, the fact that greater activity in the caudate nucleus was associated with worse and slower task performance, as opposed to better performance with greater putamen activity, was unexpected. The relationship between putamen signal and faster RTs compared with the relationship between caudate nucleus signal and slower RTs may be in line with their differential involvement in motor versus cognitive processes, respectively[6,7]. In that view, however, the involvement of caudate nucleus activity in cognitive processes would be expected to predict higher accuracy instead of lower accuracy, which we observed here. We remain puzzled about this result. It is possible that higher caudate nucleus activity improves accuracy on reversal trials when cognitive control is required to update the correct response, but hampers performance on regular trials when the quick, learned response is correct. Given the many regular trials compared with reversal trials, this could surface as the observed negative main effect of caudate nucleus signal on accuracy across all trials. However, it would also suggest a positive interaction effect of unexpected versus expected outcomes. While the estimate of

that effect was positive, it was not significant. Although not significant, the direction of the interaction effects between caudate nucleus signal and outcome expectancy and valence may be consistent with the hypothesized tonic versus phasic mechanism of drug action. Methylphenidate-induced increases of tonic dopamine levels in lower dopamine synthesis participants might increase their salience signaling to unexpected outcomes while also reducing the effectiveness of phasic dopamine release in response to unexpected rewards compared with punishments. However, given the exploratory nature of these analyses and the fact that the interaction effects were not significant, this remains speculation.

The current task design ensured that requirements for response shifting were matched between the two types of reversal trials. Participants needed to shift responses to the other button to indicate that their predictions had been updated after both unexpected rewards and punishments. This ensured that any valence-specific reversal comparison was not confounded by differences in processing related to motor preparation or response perseveration. However, an important implication of this design is that a shift towards better punishment versus reward-based reversal might reflect one of two processes. First, it might reflect better punishment surprise coding. Second, it might reflect a greater tendency to adopt a canonical win-stay/lose-shift strategy: in most (instrumental) contexts, where outcomes are contingent on actions, punishments signal the need to shift behavior. Notably, this was not the case in the current stimulus-outcome prediction task. In fact, a win-stay/lose-shift strategy was inappropriate selectively after unexpected rewards.

One possibility is that the pattern of drug effects reflects an effect of striatal dopamine on the reliance on a canonical win-stay/lose-shift strategy. However, we found no evidence for the adoption of such a strategy on the regular non-reversal trials, and no modulation by drugs or synthesis capacity, suggesting that this is unlikely to be the case.

It might be noted that a previous study with the same paradigm had revealed a positive rather than a negative link between striatal BOLD signal and dopamine-related boosting of reward versus punishment learning[3]. We remain puzzled about this, but emphasize that the locus of the drug effect in that previous study was much more ventral than the dorsomedial striatal effect observed here. This raises the possibility that participants adopted different behavioral strategies across the two experiments, relying more readily on a NoGo/switch strategy in the current experiment, but a Go-for-reward strategy in the previous experiment. This hypothesis can be tested in a future study in which Go/NoGo response demands and outcome valence are crossed by design. It is substantiated by the finding that all participants in the current study shifted responses after unexpected punishment much more readily than after unexpected reward compared with the previous study[3] (Supplementary Figure 1a).

In addition to the dopamine synthesis-dependent drug effects on reward versus punishment learning, we observed a strong main effect of methylphenidate, increasing

overall task performance irrespective of trial types, and a smaller main effect of sulpiride, decreasing overall performance (Figure 1). These non-specific performance enhancing effects of methylphenidate were accompanied by task-independent increases in BOLD signal in bilateral dorsal parietal clusters, especially for lower-dopamine participants (Supplementary Figure 9b-c; Supplementary Table 3), and might reflect nonspecific increases in general sustained attention and/or cognitive effort[8,9], often associated with activation of the dorsal attention network[10,11]. Conversely, the non-specific performance impairing effects of sulpiride were accompanied by task-nonspecific decreases in putamen signal and increases in motor cortex signal (Supplementary Figure 9d), possibly in line with an overall presynaptic disinhibition of dopamine release, leading to decreased striatal indirect pathway activity and disinhibition of motor responding, and resulting in a numerical, albeit not significant, overall decrease in response times.

Supplementary Methods

Performance-based participant exclusion

There was a minimal task performance criterion of having at least 20 reversals per session. Fewer than 20 reversal trials occurring in a session was considered a sign of task disengagement. To test this, we correlated the number of reversals in a session with the number of missed trials in that session, across all available sessions (i.e. without any exclusions). This revealed a strong negative correlation ($t_{(291)} = -17.49$, $p < 2.2e-16$), indicating that those sessions on which participants had few reversal trials also contained a large number of missed trials (rather than simply incorrect trials), presumably reflecting task disengagement. Supplementary Figure 15 illustrates this correlation and shows that the preset performance criterion of 20 reversals resulted in the exclusion of sessions with many missed trials.

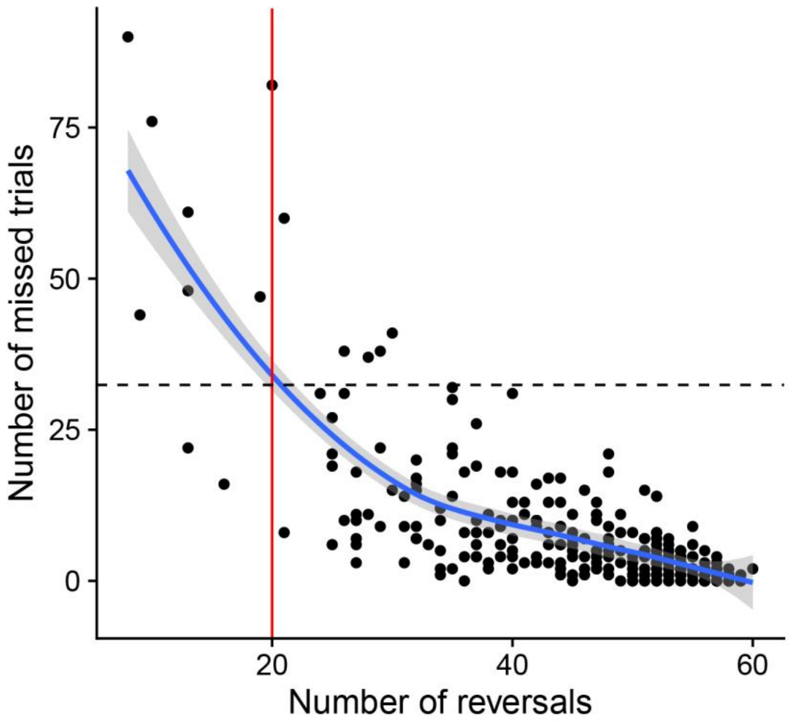

Supplementary Figure 15. ***Relationship between the number of reversals in a session (a marker of overall task performance) and the number of missed trials in that session.*** *Sessions with few reversals generally contained a large number of missed trials. N=99 participants. Vertical red line indicates the cutoff for the minimum number of reversals required per session for inclusion. Horizontal dashed line indicates the mean + 2 standard deviations of the number of missed trials. Error band displays the 95% confidence interval of fitted line. Source data are provided with this paper.*

PET acquisition and preprocessing

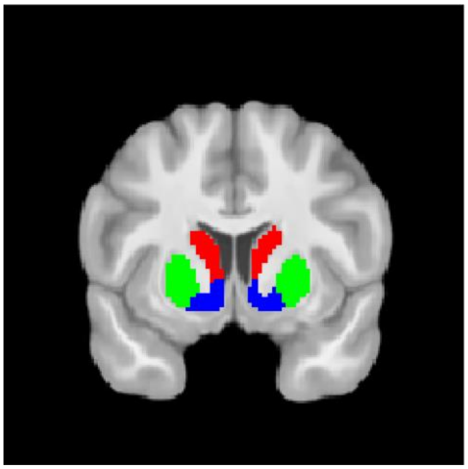

Supplementary Figure 16. ***Analysis masks of the striatal regions of interest: caudate nucleus (red), putamen (green), and nucleus accumbens (ventral striatum; blue).*** *The masks are based on an independent, functional connectivity-based parcellation of the striatum (ref 12), and are overlaid on the group-average T1-weighted anatomical scan in MNI152 coordinate space.*

fMRI preprocessing details

All MRI data were preprocessed using fMRIPrep (1.2.6-1; RRID:SCR_016216;[13,14]), which is based on Nipype (1.1.7; RRID:SCR_002502;[15,16]). Many internal operations of fMRIPrep use Nilearn (0.5.0; RRID:SCR_001362l;[17]), mostly within the functional processing workflow. For more details of the pipeline, see the section corresponding to workflows in fMRIPrep's documentation.

Anatomical data preprocessing. The T1-weighted (T1w) image was corrected for intensity non-uniformity (INU) using `N4BiasFieldCorrection` (ANTs 2.2.0)[18], and used as T1w-reference throughout the workflow. The T1w-reference was then skull-stripped using `antsBrainExtraction.sh` with OASIS as target template. Brain surfaces were reconstructed using `recon-all` (FreeSurfer 6.0.1, RRID:SCR_001847;[19]), and the brain mask estimated previously was refined with a custom variation of the method to reconcile ANTs-derived and FreeSurfer-derived segmentations of the cortical gray-matter of Mindboggle (RRID:SCR_002438;[20]). Spatial normalization to the ICBM 152 Nonlinear Asymmetrical template version 2009c (RRID:SCR_008796;[21]) was performed through nonlinear registration with `antsRegistration` (RRID:SCR_004757;[22]), using brain-extracted versions of both T1w volume and template. Brain tissue segmentation of cerebrospinal fluid (CSF), white-matter (WM) and gray-matter (GM) was performed on the brain-extracted T1w using `fast` (FSL 5.0.9, RRID:SCR_002823;[23]).

Functional data preprocessing. Before preprocessing the functional runs with fMRIPrep, we combined the multi-echo data into single time-series per run with the multi-echo toolbox (https://github.com/Donders-Institute/multiecho; commit Nr.: 9356bc51ef) using the TE algorithm, in which the different echoes are weighted by their echo time. The multi-echo combined runs were then preprocessed further with fMRIPrep.

For each of the BOLD runs found per subject, the following preprocessing was performed. First, a reference volume and its skull-stripped version were generated using a custom methodology of fMRIPrep. The BOLD reference was then co-registered to the T1w reference using `bbregister` (FreeSurfer) which implements boundary-based registration[24]. Co-registration was configured with nine degrees of freedom to account for distortions remaining in the BOLD reference. Head-motion parameters with respect to the BOLD reference (transformation matrices, and six corresponding rotation and translation parameters) were estimated before any spatiotemporal filtering using `mcflirt` (FSL 5.0.9,[25]). BOLD runs were slice-time corrected using `3dTshift` from AFNI 20160207 (RRID:SCR_005927;[26]). The BOLD time-series (including slice-timing correction) were resampled onto their original, native space by applying a single, composite transform to correct for head-motion and susceptibility distortions. First, a reference volume and its skull-stripped version were generated using a custom methodology of fMRIPrep. The BOLD

time-series were resampled to MNI152NLin2009cAsym standard space. Automatic removal of motion artifacts using independent component analysis (ICA-AROMA[27]) was performed on the preprocessed BOLD time-series in MNI space after removal of non-steady state volumes and spatial smoothing with an isotropic, Gaussian kernel of 6 mm FWHM (full-width at half-maximum). The motion artifacts were collected as noise-regressors. Several confounding time-series were calculated based on the preprocessed BOLD: framewise displacement (FD), DVARS and three region-wise global signals. FD and DVARS were calculated for each functional run, both using their implementations in Nipype (following the definitions by[28]). The three global signals were extracted within the CSF, the WM, and the whole-brain masks. Additionally, a set of physiological regressors were extracted to allow for component-based noise correction (CompCor[29]). Principal components were estimated after high-pass filtering the preprocessed BOLD time-series (using a discrete cosine filter with 128s cut-off) for the two CompCor variants: temporal (tCompCor) and anatomical (aCompCor). Six tCompCor components were then calculated from the top 5% variable voxels within a mask covering the subcortical regions. This subcortical mask was obtained by heavily eroding the brain mask, which ensured it did not include cortical GM regions. For aCompCor, six components were calculated within the intersection of the aforementioned mask and the union of CSF and WM masks calculated in T1w space, after their projection to the native space of each functional run (using the inverse BOLD-to-T1w transformation). All resamplings could be performed with a single interpolation step by composing all the pertinent transformations (i.e. head-motion transform matrices, susceptibility distortion correction when available, and co-registrations to anatomical and template spaces). Gridded (volumetric) resamplings were performed using `antsApplyTransforms` (ANTs), configured with Lanczos interpolation to minimize the smoothing effects of other kernels[30]. Non-gridded (surface) resamplings were performed using `mri_vol2surf` (FreeSurfer).

fMRI quality assurance procedure

We assessed the quality of the individual fMRI datasets before including them in the group analyses, using first-level contrasts of non-interest and quality reports created using custom Matlab code (https://github.com/bramzandbelt/fmri_preprocessing_and_qa_code) with SPM8 in Matlab R2015a. We first judged each drug session's unthresholded contrast map of activity related to button presses. If the activation was deemed sufficient the session was included in the group analyses. If it was not, we checked the contrast map for unexpected versus expected outcomes. If there was not sufficient activation in that contrast either we searched for low tSNR values (<80) or artifacts in the quality reports that might explain the lack of activation. When those were present the session was excluded from the analyses, but the session was included if there was no obvious explanation for the lack of activation in the reports.

PPI analysis

We performed a generalized PPI (gPPI) analysis to test for task condition-dependent temporal correlation, a measure interpretable as functional connectivity, between BOLD signal in seed regions in the right caudate nucleus and in the individually defined FFA/PPA regions of interest. The significant cluster in the right caudate nucleus showing an effect of methylphenidate on surprising outcome-related signal (Figure 2b) was selected as seed region of the PPI analysis of contrasts involving methylphenidate, and the right caudate nucleus cluster with a significant sulpiride effect (Figure 2c) was selected for the PPI analysis involving sulpiride. The gPPI analysis was performed using the gPPI toolbox for SPM[31].

First, the first eigenvariate of the time series of activity in the seed region was extracted and adjusted for an F-contrast of all task regressors of interest. The extracted time series was used to create new PPI regressors representing the interaction between the seed region's activity and each of the task regressors. These regressors were then added to a new GLM, together with the regressors of the regular fMRI analyses. Voxel-wise estimation of the new GLM tested how much each voxel's activity was related to activity in the seed region under each task condition. Like before, we contrasted task regressors of outcomes associated with the face stimulus with those associated with the scene stimulus, but now using the PPI regressors. The resulting contrast estimates were extracted from the individually defined FFA and PPA ROIs, and a stimulus-specificity index was created by subtracting the estimates in the PPA from those in the FFA (FFA minus PPA). Greater values of this index indicated greater functional connectivity between the seed region and the FFA or PPA during outcomes associated with the face or scene stimulus, respectively. Finally, the contrast estimates were analyzed in a repeated-measures ANOVA, including dopamine synthesis capacity in the caudate nucleus as a covariate.

Analysis of trial-level BOLD signal

To investigate how changes in BOLD signal contributed to task performance, we added trial-by-trial estimates of the BOLD response as independent variables to the Bayesian mixed-effects models of behavior.

For this analysis, we used BOLD signal from the clusters where there were significant drug effects of interest. These were the aPFC cluster with a dopamine synthesis-dependent methylphenidate effect on unexpected reward versus punishment signal (Figure 4a; 46 voxels), the putamen clusters with a synthesis-independent methylphenidate effect on surprising outcome-related signal (Figure 2a; 101 voxels), the two clusters in the right caudate nucleus with dopamine synthesis-dependent effects of methylphenidate and sulpiride on surprising outcome-related signal (Figure 2b-c; 18 and 15 voxels for

methylphenidate's and sulpiride's cluster, respectively), and the stimulus-specificity index of BOLD signal in the individually defined FFA/PPA. Additionally, we included trial-level BOLD signal from the bilateral cluster in the supramarginal gyrus where there was a dopamine synthesis-independent main effect of methylphenidate (Supplementary Table 3; 124 voxels) to use as a control region.

First, we extracted the first eigenvariate of the BOLD time-series (corrected for confound regressors) from each cluster, using SPM12. Then, we up-sampled the time-series with a factor of 23 to a TR of 100 ms and divided the series into 8-second-long epochs, each starting at the onset of a trial's outcome. Finally, we fit the canonical SPM hemodynamic response function (HRF) to the BOLD data, using separate GLMs for each epoch, to obtain trial-by-trial estimates of the BOLD amplitude to the trials' outcomes. Note that epochs of 8 seconds were long enough to include the peak of the HRF, but this caused the end of the epoch to also include the onset of the outcome of the next trial. However, given the sluggishness of the BOLD response, signal buildup related to the next trial's outcome at the end of the epoch would have had a relatively small contribution to the overall signal in the epoch. The settings and custom Matlab code for these procedures were based on the Temporal Analysis of fMRI data Toolbox (https://github.com/tuhauser/TAfT)[32].

Having obtained trial-by-trial BOLD estimates for each participant, we estimated separate trial-wise mixed-effects models of behavior for each cluster with the BOLD estimates as a predictor, both as a main effect and in interaction with response update type and valence in the fixed and random effects. The fMRI predictor was scaled and mean-centered, and it was lagged by one trial, so the model assessed the effect of the BOLD response to the previous outcome on behavior on the current trial. The main effect tests whether the brain activation to an outcome has an effect on the performance on the subsequent trial, and the interactions test whether that effect differs with trial type.

Brain-behavior correlations

In addition to the mixed-effects models with trial-level BOLD signal, we assessed the association between individual contrast estimates from the clusters with significant group-level drug effects of interest and model coefficients of performance. To this end, we extracted the individual contrast estimates (custom Matlab code) and performed Pearson correlation analyses (in R) with the model coefficients for the respective drug effects on overall accuracy/RT, reversal versus regular accuracy/RT, and reward versus punishment reversal accuracy/RT (Supplementary Table 6).

Supplementary References

1. Cools, R., Clark, L., Owen, A. M. & Robbins, T. W. Defining the Neural Mechanisms of Probabilistic Reversal Learning Using Event-Related Functional Magnetic Resonance Imaging. *Journal of Neuroscience* **22**, 4563–4567 (2002).

2. Robinson, O. J., Frank, M. J., Sahakian, B. J. & Cools, R. Dissociable responses to punishment in distinct striatal regions during reversal learning. *NeuroImage* **51**, 1459–1467 (2010).

3. van der Schaaf, M. E. *et al.* Establishing the Dopamine Dependency of Human Striatal Signals During Reward and Punishment Reversal Learning. *Cerebral Cortex* **24**, 633–642 (2014).

4. Kumakura, Y. *et al.* Age-dependent decline of steady state dopamine storage capacity of human brain: An FDOPA PET study. *Neurobiology of Aging* **31**, 447–463 (2010).

5. Cools, R. *et al.* Striatal Dopamine Predicts Outcome-Specific Reversal Learning and Its Sensitivity to Dopaminergic Drug Administration. *The Journal of Neuroscience* **29**, 1538–1543 (2009).

6. Haber, S. N. & Knutson, B. The Reward Circuit: Linking Primate Anatomy and Human Imaging. *Neuropsychopharmacology* **35**, 4–26 (2010).

7. Joel, D. & Weiner, I. The connections of the dopaminergic system with the striatum in rats and primates: An analysis with respect to the functional and compartmental organization of the striatum. *Neuroscience* **96**, 451–474 (2000).

8. Hofmans, L. *et al.* Methylphenidate boosts choices of mental labor over leisure depending on striatal dopamine synthesis capacity. *Neuropsychopharmacology* **45**, 2170–2179 (2020).

9. Westbrook, A. *et al.* Dopamine promotes cognitive effort by biasing the benefits versus costs of cognitive work. *Science* **367**, 1362–1366 (2020).

10. Roberts, C. A., Jones, A., Sumnall, H., Gage, S. H. & Montgomery, C. How effective are pharmaceuticals for cognitive enhancement in healthy adults? A series of meta-analyses of cognitive performance during acute administration of modafinil, methylphenidate and D-amphetamine. *European Neuropsychopharmacology* **38**, 40–62 (2020).

11. Tomasi, D. *et al.* Methylphenidate enhances brain activation and deactivation responses to visual attention and working memory tasks in healthy controls. *NeuroImage* **54**, 3101–3110 (2011).

12. Piray, P. *et al.* Dopaminergic Modulation of the Functional Ventrodorsal Architecture of the Human Striatum. *Cerebral Cortex* **27**, 485–495 (2017).

13. Esteban, O. *et al.* fMRIPrep: A robust preprocessing pipeline for functional MRI. *Nature Methods* **16**, 111–116 (2019).

14. Esteban, O. *et al.* fMRIPrep: A robust preprocessing pipeline for functional MRI. Zenodo https://doi.org/10.5281/zenodo.3876458 (2020).

15. Esteban, O. *et al.* Nipy/nipype: 1.5.0. Zenodo https://doi.org/10.5281/zenodo.3874968 (2020).

16. Gorgolewski, K. *et al.* Nipype: A Flexible, Lightweight and Extensible Neuroimaging Data Processing Framework in Python. *Frontiers in Neuroinformatics* **5**, (2011).

17. Abraham, A. *et al.* Machine learning for neuroimaging with scikit-learn. *Frontiers in Neuroinformatics* **8**, (2014).

18. Tustison, N. J. *et al.* N4ITK: Improved N3 Bias Correction. *IEEE Transactions on Medical Imaging* **29**, 1310–1320 (2010).

19. Dale, A. M., Fischl, B. & Sereno, M. I. Cortical Surface-Based Analysis: I. Segmentation and Surface Reconstruction. *NeuroImage* **9**, 179–194 (1999).

20. Klein, A. *et al.* Mindboggling morphometry of human brains. *PLOS Computational Biology* **13**, e1005350 (2017).

21. Fonov, V., Evans, A., McKinstry, R., Almli, C. & Collins, D. Unbiased nonlinear average age-appropriate brain templates from birth to adulthood. *NeuroImage* **47**, S102 (2009).

22. Avants, B. B., Epstein, C. L., Grossman, M. & Gee, J. C. Symmetric diffeomorphic image registration with cross-correlation: Evaluating automated labeling of elderly and neurodegenerative brain. *Medical Image Analysis* **12**, 26–41 (2008).

23. Zhang, Y., Brady, M. & Smith, S. Segmentation of brain MR images through a hidden Markov random field model and the expectation-maximization algorithm. *IEEE Transactions on Medical Imaging* **20**, 45–57 (2001).

24. Greve, D. N. & Fischl, B. Accurate and robust brain image alignment using boundary-based registration. *NeuroImage* **48**, 63–72 (2009).

25. Jenkinson, M., Bannister, P., Brady, M. & Smith, S. Improved Optimization for the Robust and Accurate Linear Registration and Motion Correction of Brain Images. *NeuroImage* **17**, 825–841 (2002).

26. Cox, R. W. & Hyde, J. S. Software tools for analysis and visualization of fMRI data. *NMR in Biomedicine* **10**, 171–178 (1997).

27. Pruim, R. H. R. *et al.* ICA-AROMA: A robust ICA-based strategy for removing motion artifacts from fMRI data. *NeuroImage* **112**, 267–277 (2015).

28. Power, J. D. *et al.* Methods to detect, characterize, and remove motion artifact in resting state fMRI. *NeuroImage* **84**, 320–341 (2014).

29. Behzadi, Y., Restom, K., Liau, J. & Liu, T. T. A component based noise correction method (CompCor) for BOLD and perfusion based fMRI. *NeuroImage* **37**, 90–101 (2007).

30. Lanczos, C. Evaluation of Noisy Data. *Journal of the Society for Industrial and Applied Mathematics: Series B, Numerical Analysis* **1**, 76–85 (1964).

31. McLaren, D. G., Ries, M. L., Xu, G. & Johnson, S. C. A generalized form of context-dependent psychophysiological interactions (gPPI): A comparison to standard approaches. *NeuroImage* **61**, 1277–1286 (2012).

32. Hauser, T. U. *et al.* Temporally Dissociable Contributions of Human Medial Prefrontal Subregions to Reward-Guided Learning. *Journal of Neuroscience* **35**, 11209–11220 (2015).
