# Supplementary material for: Striatal dopamine dissociates methylphenidate effects on value-based versus surprise-based reversal learning
Source: Nat Commun. 2022 Aug 24;13:4962. doi: 10.1038/s41467-022-32679-1 (PMC9402573; doi:10.1038/s41467-022-32679-1)
Supplement: Supplementary file 3 — Reporting Summary [file 41467_2022_32679_MOESM3_ESM.pdf]

nature portfolio

Corresponding author(s): Ruben van den Bosch

Last updated by author(s): Feb 25, 2022

Reporting Summary

Nature Portfolio wishes to improve the reproducibility of the work that we publish. This form provides structure for consistency and transparency in reporting. For further information on Nature Portfolio policies, see our Editorial Policies and the Editorial Policy Checklist.

Statistics

For all statistical analyses, confirm that the following items are present in the figure legend, table legend, main text, or Methods section.

n/a	Confirmed	
☐	☒	The exact sample size (*n*) for each experimental group/condition, given as a discrete number and unit of measurement
☐	☒	A statement on whether measurements were taken from distinct samples or whether the same sample was measured repeatedly
☐	☒	The statistical test(s) used AND whether they are one- or two-sided<br>*Only common tests should be described solely by name; describe more complex techniques in the Methods section.*
☐	☒	A description of all covariates tested
☐	☒	A description of any assumptions or corrections, such as tests of normality and adjustment for multiple comparisons
☐	☒	A full description of the statistical parameters including central tendency (e.g. means) or other basic estimates (e.g. regression coefficient) AND variation (e.g. standard deviation) or associated estimates of uncertainty (e.g. confidence intervals)
☐	☒	For null hypothesis testing, the test statistic (e.g. *F*, *t*, *r*) with confidence intervals, effect sizes, degrees of freedom and *P* value noted<br>*Give P values as exact values whenever suitable.*
☐	☒	For Bayesian analysis, information on the choice of priors and Markov chain Monte Carlo settings
☐	☒	For hierarchical and complex designs, identification of the appropriate level for tests and full reporting of outcomes
☐	☒	Estimates of effect sizes (e.g. Cohen's *d*, Pearson's *r*), indicating how they were calculated

Our web collection on statistics for biologists contains articles on many of the points above.

Software and code

Policy information about availability of computer code

Data collection	Presentation (Neurobehavioral Systems; version 18.3 03.11.16)
Data analysis	All code available at https://doi.org/10.34973/bc23-mz79<br>Software:<br>R (4.0.2), Matlab (2018b), Python3.7<br>fMRIPREP (version 1.2.6-1)<br>multi-echo toolbox (https://github.com/Donders-Institute/multiecho; commit Nr.: 9356bc51ef)<br>Statistical Parametric Mapping 12 (SPM12; https://www.fil.ion.ucl.ac.uk/spm/software/spm12/)<br>FSL (version 6.0.1)<br>slice_display toolbox (https://github.com/bramzandbelt/slice_display)<br>generalized PPI (gPP) toolbox (https://www.nitrc.org/projects/gppi)<br>tidyverse R package (version 1.3.1)<br>`brms` R package (version 2.14.0)<br>`afex` R package (version 0.28-0)

For manuscripts utilizing custom algorithms or software that are central to the research but not yet described in published literature, software must be made available to editors and reviewers. We strongly encourage code deposition in a community repository (e.g. GitHub). See the Nature Portfolio guidelines for submitting code & software for further information.

Data

Policy information about availability of data

All manuscripts must include a data availability statement. This statement should provide the following information, where applicable:

- Accession codes, unique identifiers, or web links for publicly available datasets
- A description of any restrictions on data availability
- For clinical datasets or third party data, please ensure that the statement adheres to our policy

The minimally processed data used in this study and the overarching project it is part of are available from the Donders Institute Data Repository (https://doi.org/10.34973/wn51-ej53). The final data derivatives relevant to the current work are available from a separate collection on the Donders Institute Data Repository (https://doi.org/10.34973/bc23-mz79).

Field-specific reporting

Please select the one below that is the best fit for your research. If you are not sure, read the appropriate sections before making your selection.

☐ Life sciences ☒ Behavioural & social sciences ☐ Ecological, evolutionary & environmental sciences

For a reference copy of the document with all sections, see nature.com/documents/nr-reporting-summary-flat.pdf

Behavioural & social sciences study design

All studies must disclose on these points even when the disclosure is negative.

Study description	Quantitative; within-subject, placebo-controlled, double-blind cross-over design.
Research sample	Representative sample of 100 healthy human participants, 50% male, 50% female. Age: range 18 to 43, mean (SD) = 23.0 (5.0) years. The sample size in the current work was determined by the number of participants included in the overarching project that this study is part of, rather than with an a priori power calculation for the specific task reported here.
Sampling strategy	Our sample size estimation of the overarching project that the current manuscript is part of was calculated based on the effect size of a previous pharmacological-behavioural study recently performed by our group (CMO Arnhem-Nijmegen protocol 2013/568). In that study, 95 participants received placebo and MPH on two sessions and performed a series of cognitive tasks. The effect size in that study was r=0.30 (p<0.001, rank correlation). Here we used G*power3 software for power calculation, which demonstrated that using a multiple linear regression model with 7 predictor variables (7 task outcomes in overarching project) an effect-size of f2 = 0.1 (multiple regression equivalent of r=0.3) may be detected with 85% power from a sample size of 92 subjects (pre-defined p value = 5%). We rounded this number up to 100 to account for potential drop-outs and technical problems.
Data collection	Task response data were collected on a computer (Windows 7 Enterprise OS, on a DELL PRECISION T3500) using an MRI compatible response pad (Current Designs, Inc; Philadelphia, PA, USA) in the MRI scanner (3T Siemens Magnetom Skyra MRI scanner). PET data were acquired on a state-of-the-art PET/CT scanner (Siemens Biograph mCT; Siemens Medical Systems, Erlangen, Germany).<br>During task performance, the MRI scanner was operated by an experimenter; during the PET scan, medical personnel was present to administer the radiotracer and operate the PET scanner.<br>Drug administration was double-blind, and only unblinded to the experimenters after completion of all data collection.
Timing	January 2017 - October 2018
Data exclusions	Six participants were excluded due to failure to reach task performance criterion on one of the three task sessions. The criterion was a minimum of 20 reversal trials. This criterion was determined when data collection was already ongoing, but before drug status deblinding.
Non-participation	Six participants dropped out because of discomfort in the MRI or PET scanner (N=4), personal reasons (N=1) or technical failure of the PET scanner (N=1).
Randomization	Participants were randomly assigned the order in which they received the two drugs and placebo.

Reporting for specific materials, systems and methods

We require information from authors about some types of materials, experimental systems and methods used in many studies. Here, indicate whether each material, system or method listed is relevant to your study. If you are not sure if a list item applies to your research, read the appropriate section before selecting a response.

Materials & experimental systems

n/a	Involved in the study
☒	☐ Antibodies
☒	☐ Eukaryotic cell lines
☒	☐ Palaeontology and archaeology
☒	☐ Animals and other organisms
☐	☒ Human research participants
☒	☐ Clinical data
☒	☐ Dual use research of concern

Methods

n/a	Involved in the study
☒	☐ ChIP-seq
☒	☐ Flow cytometry
☐	☒ MRI-based neuroimaging

Human research participants

Policy information about studies involving human research participants

Population characteristics

One hundred healthy volunteers, 50 women and 50 men, were recruited for the study (age at inclusion: range 18 to 43, mean (SD) = 23.0 (5.0) years).
All participants had Dutch as native language and were right-handed.
Exclusion criteria included any current or previous psychiatric or neurological disorders, having a first-degree family member with a current or previous psychiatric disorder, clinically significant hepatic, cardiac, renal, metabolic or pulmonary disease, epilepsy, hyper or hypotension, habitual smoking or drug use, pregnancy, and MRI contraindications, such as unremovable metal parts in the body and claustrophobia.

Recruitment

People were recruited via an advertisement on the Radboud University electronic database for research participants, and via advertisement flyers around Nijmegen.
The majority of participants in the electronic database are higher education students from around Nijmegen. In combination with the fact that this was a large multi-session study to participate in, this may have caused a selection bias towards motivated participants (which may relate to dopamine levels).

Ethics oversight

The study was approved by the local ethics committee ("Commissie Mensgebonden Onderzoek", CMO region Arnhem-Nijmegen, The Netherlands: protocol NL57538.091.16).

Note that full information on the approval of the study protocol must also be provided in the manuscript.

Magnetic resonance imaging

Experimental design

Design type

Task; event-related

Design specifications

Per subject: 3 blocks of 119 trials, resulting in a total of 357 trials per session (1071 total over three sessions). Trial length: 4500 ms; Intertrial interval: 1000 - 3500 ms.

Behavioral performance measures

Button presses (two possible) and response times were recorded. If too many subsequently missed or incorrect trials occurred, the task halted automatically to allow the experimenter to check on the participant and provide instructions or fix a problem if necessary.

Acquisition

Imaging type(s)

functional, structural

Field strength

3 Tesla

Sequence & imaging parameters

Functional: 32-channel head coil. Images with blood-oxygen level-dependent (BOLD) contrast were acquired in 3 runs, using a whole-brain T2*-weighted gradient echo multi-echo echo planar imaging (EPI) sequence (38 slices per volume; interleaved slice acquisition; repetition time, 2320 ms; echo times, 9 ms, 19.3 ms, 30 ms, and 40 ms; field of view: 211x211 mm; flip angle 90°; 64x64 matrix; 3.3mm in-plane resolution; 2.5mm slice thickness, 0.4mm slice gap).

Structural: T1-weighted magnetization prepared, rapid-acquisition gradient echo sequence (192 sagittal slices; repetition time, 2300 ms; echo time, 3.03 ms; field of view: 256x256mm; flip angle, 8°; 256x256 matrix; 1.0mm in-plane resolution; 1.0mm slice thickness).

Area of acquisition

whole brain

Diffusion MRI ☐ Used ☒ Not used

Preprocessing

Preprocessing software

multi-echo data were combined into a single time-series per fMRI run with the multi-echo toolbox (https://github.com/

Preprocessing software	Donders-Institute/multiecho; commit Nr.: 9356bc51ef) using the TE algorithm.<br>fMRIPrep (1.2.6-1; RRID:SCR_016216) with slice time correction. ICA-AROMA noise components were not removed but used in first-level regression models. Data were smoothed with a 6 mm full-width-at-half-maximum kernel.
Normalization	Spatially normalized to MNI152 space with fMRIPrep.
Normalization template	MNI152NLin2009cAsym in fMRIPrep
Noise and artifact removal	We assessed the quality of the individual fMRI datasets before including them in the group analyses, using first-level contrasts of non-interest and quality reports created using custom Matlab code (https://github.com/bramzandbelt/fmri_preprocessing_and_qa_code) with SPM8 in Matlab R2015a. We first judged each drug session's unthresholded contrast map of activity related to button presses. If the activation was deemed sufficient the session was included in the group analyses. If it was not, we checked the contrast map for unexpected versus expected outcomes. If there was not sufficient activation in that contrast either we searched for low tSNR values (<80) or artifacts in the quality reports that might explain the lack of activation. When those were present the session was excluded from the analyses, but the session was included if there was no obvious explanation for the lack of activation in the reports.
Volume censoring	No volume censoring.

Statistical modeling & inference

Model type and settings	mass univariate, fixed effect. First-level: 6 realignment parameters, framewise displacement, global CSF and global white matter signal, 6 anatomical principal component noise regressors (aCompCor), and all independent components labeled as noise by ICA-AROMA (different number for each fMRI run). Low-frequency drifts in the data were controlled using a high-pass filter with a 128 s cutoff.
Effect(s) tested	For each drug separately, we created contrasts for the effects of expectancy ([unexpected reward - expected reward] + [unexpected punishment - expected punishment]), valence ([unexpected reward - unexpected punishment] + [expected reward - expected punishment]), and the expectancy x valence interaction ([unexpected reward - expected reward] - [unexpected punishment - expected punishment]). The contrasts for the drug effects were created by subtracting the contrast vectors for placebo from the contrast vectors for methylphenidate/sulpiride. The resulting contrast images of each participant were taken to the group-level analysis and submitted to a one-sample t-test, with participants' striatal dopamine synthesis capacity values as covariate

Specify type of analysis: ☐ Whole brain ☐ ROI-based ☒ Both

Anatomical location(s)	Combination of caudate nucleus, putamen and nucleus accumbens ROI masks, based on an independent, functional connectivity-based parcellation of the striatum (Piray et al., 2017. Cereb Cortex).
Statistic type for inference<br>(See Eklund et al. 2016)	Voxel-wise in combined ROI mask
Correction	$p < 0.05$ peak-level FWE, after small volume correction for combined striatum ROIs masks

Models & analysis

n/a	Involved in the study
☐	☒ Functional and/or effective connectivity
☒	☐ Graph analysis
☒	☐ Multivariate modeling or predictive analysis

Functional and/or effective connectivity	psychophysiological interaction (PPI)
